# Respiratory health and its determinants among Nunavimmiut: results from the *Qanuilirpitaa*? 2017 Nunavik Health Survey

**DOI:** 10.17269/s41997-022-00722-9

**Published:** 2023-01-09

**Authors:** Philippe Robert, Benoît Lévesque, Jean Bourbeau, Faiz Ahmad Khan, Louis-Philippe Boulet, Marc-André Dubé, Jean-François Proulx, Pierre Ayotte

**Affiliations:** 1 Institut national de santé publique du Québec, Quebec City, QC Canada; 2 Département de médecine sociale et préventive, Université Laval, Quebec City, QC Canada; 3 Axe santé des populations et pratiques optimales en santé, Centre de recherche du CHU de Québec, Quebec City, QC Canada; 4 Respiratory Epidemiology and Clinical Research Unit, Department of Medicine, McGill University, Montreal, QC Canada; 5 Research Institute, McGill University Health Centre, Montreal Chest Institute, Montreal, QC Canada; 6 McGill International TB Centre, Montreal, QC Canada; 7 Institut universitaire de cardiologie et de pneumologie de Québec, Quebec City, QC Canada; 8 Département de médecine, Université Laval, Quebec City, QC Canada; 9 Department of Public Health, Nunavik Regional Board of Health and Social Services, Kuujjuaq, QC Canada

**Keywords:** Inuit, Indigenous peoples, Lung disease, Pulmonary disease, chronic obstructive, Asthma, Airway obstruction, Inuit, Autochtones, maladie pulmonaire, maladie pulmonaire obstructive chronique, asthme, obstruction bronchique

## Abstract

**Objectives:**

Respiratory diseases are the leading cause of hospitalization in Nunavik (northern Québec, Canada) and contribute to disparities in life expectancy with the rest of Canada. As part of *Qanuilirpitaa?* 2017, a cross-sectional population-based health survey, we sought to describe the prevalence of respiratory health indicators, including the first estimate of airway obstruction based on spirometry in an Inuit population, and explore their associated characteristics.

**Methods:**

We analyzed data from 1296 participants aged 16 years and older, using multivariate logistic regression to assess characteristics associated with spirometry-determined airway obstruction and self-reported respiratory symptoms, i.e., wheezing in the last year and chronic cough during at least 3 months.

**Results:**

In this relatively young population (83% aged 16 to 54), the prevalences of wheezing, chronic cough, and airway obstruction were, respectively, 27% (95% CI 24–30), 21% (18–23), and 17% (14–20). These estimates are prone to biases due to the relatively low participation rate (about 37%). The most consistent associations were with smoking (≥ 15 pack-years; odds ratio [OR] 3.13, 3.39, and 2.86 for the three indicators, respectively) and food security (OR 0.55 with wheezing and OR 0.26 with chronic cough), as defined in the Household Food Security Survey Module. Wheezing was also associated with allergic sensitization to dogs (2.60) and obesity (2.18). Chronic cough was associated with respiratory infections during childhood (2.12), housing in need of major repairs (1.72), and housing crowding (1.50), and was negatively associated with participation to traditional activities (0.62) and going on the land (0.64). Airway obstruction was associated with being underweight (3.84) and post-secondary education (0.40). Among young adults and women, wheezing was also associated with any inhalation of solvents for recreational purposes during their lifetime (2.62 and 1.56, respectively), while airway obstruction was associated with regular marijuana use (2.22 and 1.84, respectively).

**Conclusion:**

Smoking and food insecurity are both highly prevalent and strongly associated with respiratory symptoms in Nunavik. Together with essential smoking prevention and cessation programs, our findings suggest that solving food security and housing crises, improving socioeconomic conditions, and promoting traditional lifestyle may improve respiratory health in Nunavik.

**Supplementary Information:**

The online version contains supplementary material available at 10.17269/s41997-022-00722-9.

## Résumé

**Objectifs:**

Les maladies respiratoires sont la première cause d’hospitalisation au Nunavik (Nord-du-Québec, Canada) et contribuent aux écarts d’espérance de vie avec le reste du Canada. Dans le cadre de l’enquête transversale et populationnelle *Qanuilirpitaa?* 2017, cette étude décrit la prévalence d’indicateurs de santé respiratoire et explore les caractéristiques qui leur sont associées. Elle fournit le premier estimé de la prévalence d’obstruction respiratoire par spirométrie dans la population inuite.

**Méthodes:**

Les données de 1 296 participants âgés de 16 ans et plus ont été analysées par régression logistique multivariée pour évaluer les caractéristiques associées avec le wheezing (dans la dernière année), la toux chronique (durant au moins 3 mois) et l’obstruction bronchique (mesurée par spirométrie).

**Résultats:**

Dans cette population relativement jeune (83 % entre 16 et 54 ans), les prévalences de wheezing, de toux chronique et d’obstruction bronchique étaient de 27 % (IC95% 24-30), 21 % (18-23) et 17 % (14-20). Ces estimés pourraient être biaisés puisque le taux de participation à l’enquête était relativement faible (environ 37 %). Les associations les plus fortes et consistantes sont observées avec le tabagisme (≥ 15 paquets-années; RC 3,13, 3,39 et 2,86 pour les trois indicateurs, respectivement) et avec la sécurité alimentaire (RC 0,55 avec le wheezing et 0,26 avec la toux chronique), définie à partir du Module d’enquête sur la sécurité alimentaire des ménages. Le wheezing était notamment associé avec la sensibilisation allergique aux chiens (2,60) et l’obésité (2,18). La toux chronique était associée avec les infections respiratoires sévères dans l’enfance (2,12), un logement ayant besoin de réparations majeures (1,72) et un logement surpeuplé (1,50); tandis que participer aux activités traditionnelles (0,62) et aller souvent dans la nature (0,64) semblaient protecteurs. L’obstruction bronchique était associée avec un faible indice de masse corporelle (3,84) et un niveau de scolarité postsecondaire (0,40). Le wheezing était aussi associé avec le fait d’avoir déjà inhalé des solvants chez les jeunes adultes (2,62) et chez les femmes (1,56), tandis que l’obstruction bronchique était associée avec la consommation régulière de cannabis chez les jeunes adultes (2,22) et chez les femmes (1,84).

**Conclusion:**

Le tabagisme et l’insécurité alimentaire sont fort prévalents et fortement associés avec des symptômes respiratoires au Nunavik. En plus de rappeler l’importance de la prévention du tabagisme, ces résultats supportent la pertinence des efforts communautaires et gouvernementaux pour résoudre les crises de l’insécurité alimentaire et du logement, améliorer les conditions socioéconomiques et promouvoir la culture inuite afin d’améliorer la santé respiratoire au Nunavik.

## Introduction

Respiratory health is of particular interest to public health, since respiratory conditions are generally frequent and benefit from prevention and primary care. At the request of local public health authorities, the *Qanuilirpitaa?* 2017 health survey was set up to update information on the health status of the Inuit population of Nunavik, a region of Inuit Nunangat located north of the 55^th^ parallel in the province of Quebec. Following a consultation with major Nunavik organizations, it was decided to include a component focusing on respiratory health in the adult population, which had never been investigated in previous health surveys. Respiratory diseases are the leading cause of hospitalization and are among the main causes of death in Nunavik (NRBHSS, 2015). Respiratory diseases and infections (including lung cancer) are estimated to explain 35% of the disparity in life expectancy between Inuit and other Canadian women (8.5 years) and 15% of the disparity between Inuit and other Canadian men (9.9 years) (Peters, 2013). Excluding lung cancer, respiratory diseases and infections would explain 20% of the disparity among women and 7% of the disparity among men.

Knowing the state of respiratory health might help public health authorities and community partners to prioritize and advocate for health promotion initiatives and better access to health services. The few studies that assessed respiratory health in the Inuit population or other Indigenous populations in Canada were based on self-reported diagnosis of asthma, chronic bronchitis, or COPD (Bird et al., 2017; Bruce et al., 2014; Chang et al., 2012; Crighton et al., 2010; Garner et al., 2010; Konrad et al., 2013). These self-reported diagnoses can be influenced by recall bias and access to health care, which is often lower in remote communities. Moreover, while respiratory health has been traditionally viewed as the absence of respiratory disease, there is a new focus on the occurrence of respiratory symptoms and lung function alterations prior to the development of chronic diseases, allowing the identification of earlier determinants that are amenable to prevention (Reyfman et al., 2018). Wheezing and chronic cough were shown to be prevalent among children in Nunavut (Kovesi et al., 2011), but little is known regarding the prevalence of these symptoms in adults.

Understanding the determinants of respiratory health in Nunavik might also support health promotion initiatives, including action on the social determinants of health. While many factors are known to influence respiratory health in scientific literature, there are few empiric data about these factors in Nunavik, or in the Inuit Nunangat. In addition to tobacco smoking, which is an established risk factor for respiratory diseases across populations worldwide, some determinants may be specific to the Inuit Nunangat, given its unique environment, culture, and socioeconomic conditions.

As part of *Qanuilirpitaa? *2017, this study began with a descriptive portrait of respiratory health in Nunavik, published in a public health report (Robert et al., 2020). For convenience, we reproduced the main descriptive results in Supplementary material A (Tables A1–A7), regarding lung function, wheezing, chronic cough, chronic sputum, breathlessness, physician-diagnosed conditions (COPD, asthma, tuberculosis), respiratory medication, and allergic sensitization. The present exploratory study aims at identifying characteristics associated with respiratory health among Nunavimmiut aged 16 and older, focusing on three prevalent respiratory indicators: wheezing, chronic cough, and airway obstruction. We explored sex- and age-dependent associations with medical, lifestyle, nutritional, environmental, cultural, and social determinants of health.

## Methods

### Community engagement and ethics

The *Qanuilipirpitaa?* 2017 Nunavik Inuit Health Survey was set up following a resolution adopted by the Nunavik Regional Board of Health and Social Services (NRBHSS) requesting that a new health survey be conducted to update the information on the health status of *Nunavimmiut*. This survey was conducted in partnership with major Nunavik organizations, the *Institut national de la santé publique du Québec* and researchers from Université Laval, McGill University, and Trent University. An Inuit-led Steering Committee oversaw the preparation, conduct, data interpretation, and dissemination of the survey results. A data management committee (DMC) evaluated the usefulness of the research questions for the region, and approved data and biological sample requests. This committee included representatives from communities’ mayors, Kativik Regional Government, Makivik Corporation, Kativik Ilisarniliriniq, Avataq Cultural Institute, and Qarjuit Youth Council. The DMC met with the researchers to discuss results and provide co-interpretation of the data that takes into consideration Inuit culture and values, according to a “two-eyes approach.” Comments provided by DMC members were considered in preparing the final version of the manuscript, which was approved by the DMC. The findings were diffused to the population and partners through infographics and summaries (in Inuktitut, English, and French) and reports (in English). The *Qanuilirpitaa?* 2017 survey was also approved by the ethics committee of the *Centre de recherche du CHU de Québec*.

### Data and participants

Methods for the *Qanuilirpitaa?* 2017 Nunavik Health Survey are described in detail elsewhere (Hamel et al., 2020). The target population comprised individuals aged 16 and over whose names appeared on the Nunavik Inuit Beneficiary List provided by Makivik Corp., noninstitutionalized and living in Nunavik. To ensure a proper representation of the Nunavik population, the survey used a proportionally stratified sampling (by sex, age groups, and subregion) and survey weights (based on age, sex, home community, stratified design, and non-response). Globally among sampled individuals, the participation rate was 31% of those aged 16–30 years old and 41% of those aged 30+ years old. However, 79.7% of those contacted participated in the survey. In fact, many sampled individuals could not be contacted (because they were out of the community) or missed their appointment due to bad weather or delays in the appointment schedule. Participants were invited on board the CCGS Amundsen icebreaker for a clinical session that included physical measurements, biological sampling and a spirometry test, and an interviewer-administered validated questionnaire covering sociodemographic characteristics and lifestyle/dietary habits. Questionnaires were translated in Inuktitut by an Inuk, validated by another translator, and pretested in one community to ensure understanding. The spirometry protocol was adapted from that of the CanCOLD study (Bourbeau et al., 2014) and is comparable to the one used in the Canadian Health Measures Survey (CHMS). Briefly, spirometry was performed by experienced respiratory therapists using an EasyOne^TM^ Spirometer and following the American Thoracic Society guidelines. As in CHMS, spirometry was performed without the administration of a bronchodilator, which, for logistic reasons, was not possible in this population health survey. Participants with at least three acceptable spirometry curves (*n* = 906/1100; 82%) were included in the analysis. An experienced pneumologist examined the spirometry curves of the remaining participants and retained an additional 182 participants for which the curve could be interpreted. Pregnant participants (*n* = 30) were excluded because of the effects of pregnancy on the respiratory system.

### Respiratory health indicators

Wheezing, an indicator of asthma (To et al., 2012), was defined as “wheezing or whistling in the chest at any time during the last 12 months.” Since the word “wheezing” does not exist in Inuktitut, experienced Inuit people translated it as “  ” (“laboured breathing”) or “  ” (“whistling sound”). To verify the validity of the indicator, we tested the association of wheezing with airway obstruction, which was significant after adjustment for age and sex (odds ratio = 2.26, 95% CI [1.46–3.48]). For chronic cough, the most common epidemiologic definition was used: “cough on most days for three months each year” (Song et al., 2015). Airway obstruction—determined by spirometry—is the most frequent lung function abnormality; it serves as an epidemiologic definition of COPD among adults over 35 years old and can also indicate asthma, suboptimal lung development, or early lung decline (Eisner et al., 2010). Obstruction was defined as a FEV_1_/FVC ratio below the lower limit of normal (LLN) (Pellegrino et al., 2005). LLN was calculated according to age and sex using the NHANES III reference equation for Caucasians (Hankinson et al., 1999), since no reference equation exists for the Inuit population and the FEV_1_/FVC ratio varies little across ethnic groups (Hooper et al., 2012). In a sensitivity analysis, obstruction was defined according to the fixed ratio recommended by GOLD (< 0.7), although using this ratio underestimates obstruction in younger populations (Pellegrino et al., 2005).

### Associated characteristics

Definitions of variables and data collection tools are presented in Supplementary material B. Characteristics were selected from literature reviews about known or potential risk and protective factors of COPD, asthma, and chronic cough (Eisner et al., 2010; GOLD, 2019; GINA, 2018; Tarlo et al., 2016). Risk factors include tobacco smoking; second-hand smoke; marijuana smoking; occupational exposure to dust, gases, and fumes; respiratory infections during childhood; active tuberculosis; obesity; and lower socioeconomic status (Eisner et al., 2010; GINA, 2018; GOLD, 2019; Tarlo et al., 2016). Potential protective factors include the consumption of fruits and vegetables as well as vitamin D and omega-3 polyunsaturated fatty acid (n-3 PUFA) status (Eisner et al., 2010; GINA, 2018). n-3 PUFA are highly present in the traditional Inuit diet, more specifically in Arctic marine mammals and fishes (Baines et al., 2015). Besides typical socioeconomic indicators (education and income), we explored other social determinants of interest to the region: housing overcrowding and housing in need of repairs (NRBHSS, 2015), food insecurity (NRBHSS, 2015), and participation in traditional activities. The selection of these characteristics was discussed with and endorsed by community representatives, who also participated in data interpretation.

### Statistical analysis

Participant characteristics and respiratory indicators were described with proportions (95% confidence interval) and Wald’s chi-squared tests (Hamel et al., 2020) to compare prevalence across age groups and sexes. We conducted multivariate logistic regression to examine characteristics associated with each respiratory health indicator. Since the models are explicative, rather than predictive, we would ideally have included all potential determinants (and confounders) selected from the scientific literature, but this would have resulted in too many variables being included in the models, considering the sample size. Keeping in line with an explicative perspective, we included in final models the main confounders (age, sex, and tobacco smoking) and all variables with a *p* value < 0.2 in minimally adjusted models (for sex and age), assuming that other variables were unlikely to be confounders. Even if this strategy decreased the power compared to parsimonious models, it allowed for similar models to be compared across sexes and age groups. There was no collinearity between characteristics: all variance inflation factors (VIF) were smaller than 3.5. The statistical significance level was 0.05 (two-sided). Analyses were performed using SAS 9.4 and SAS University (SAS Institute, Cary, NC, USA), using sampling weights and SAS “survey” procedures. Variance was estimated by the Taylor series linearization method. Multiple imputation with fully conditional specification was performed to impute plausible values to missing data. This technique uses logistic regression to impute binary variables and multinomial logistic regression to impute categorical variables (White et al., 2011). Each variable was predicted with all other variables used in the study, including socioeconomic predictors and respiratory indicators as recommended by White et al. (2011). Sixty complete datasets were imputed because values for at least one variable, usually a few (median = 1; interquartile range = 0–2 variables), were missing for 57% of participants. Each imputed dataset was analyzed, then results were combined for all datasets using the “mianalyze” SAS procedure. Non-imputed and imputed descriptive data are very similar (Supplementary material C, Table C1). Outcomes were not imputed, i.e., participants were excluded from the analysis when we missed data about the outcomes (*n* = 38 for wheezing, *n* = 39 for chronic cough, *n* = 208 for airway obstruction). We used specific sampling weights for airway obstruction, to adequately represent the population.

According to an ecological framework of health, social determinants (i.e., income, education, food security, cultural determinants) presumably influence respiratory health through the mediation of other factors. For example, education does not directly influence respiratory health, but it can have an effect through smoking and other factors. While smoking should be adjusted for education status, the effect of education should not be adjusted for its mediators. Therefore, we included social determinants in the fully adjusted models, but in the text, we report their association from age/sex-adjusted models.

## Results

**Fig. 1:**
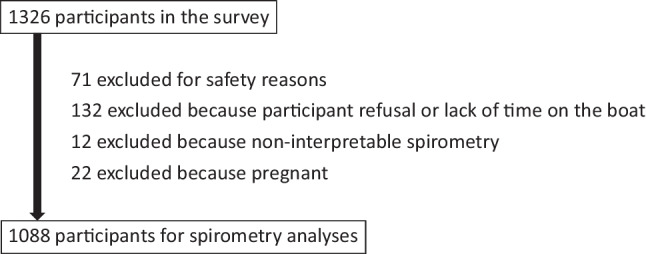
Flow chart of participants in the spirometry component

Characteristics of participants are listed in Table 1. There were 1296 participants in the study, of whom 1258 had data about wheezing, 1257 had data about chronic cough, and 1088 had spirometry data. As illustrated in Fig. 1, the lower sample size for spirometry is explained by refusal to perform the test or time constraint (132 participants), exclusion for safety reasons (71 participants), or lack of an interpretable spirometry curve (13 participants). The sample was equally divided between young adults (16–34 years) and adults (≥ 35 years), with more women than men. The most prevalent potential protective characteristics were participation in traditional activities (89%), sufficient vitamin D plasma level (68%), often going on land (44%), and marine mammal/fish consumption (≥ 7 times/week; 41%). Only a third of the population was food secure (33%). The most frequent potential risk factors were current tobacco smoking (79%), regular marijuana use (48%), second-hand smoke exposure (33%), housing crowding (32%), recreational solvent use (29%), and obesity (30%). Some variables had a higher proportion of missing data: active TB in the past (21%), personal income (14%), marine mammal and fish consumption (11%), and fruit and vegetable consumption (11%).

**Table 1:** Characteristics of participants

	Total population		16–34 years old		35 years and older		Men		Women	
	*N*	%	*N*	%	*N*	%	*N*	%	*N*	%
Women	843	48.8	422	47.7	421	49.9	–	–	–	–
Men	453	51.2	207	52.3	246	50.1	–	–	–	–
Age										
16–24 years old	364	24.9	364	54.7	–	–	130	30.3	234	25.1
25–34 years old	265	23.0	265	45.3	–	–	77	21.5	188	24.5
35–49 years old	295	24.8	–	–	295	50.3	107	24.7	188	24.8
50–59 years old	212	13.6	–	–	212	27.6	68	11.5	144	15.8
≥ 60 years old	160	10.9	–	–	160	22.2	71	12.0	89	9.8
Tobacco smoking										
Never	143	10.4	52	8.9	91	12.0	65	12.5	78	8.2
Former	149	10.7	48	7.4	101	14.1	53	10.4	96	11.0
Current < 15 pack-years	713	54.0	500	76.4	214	31.0	202	47.8	511	60.5
Current ≥ 15 pack-years	291	24.9	29	7.3	262	43.0	133	29.3	158	20.3
Electronic cigarette: in past year	145	12.2	105	17.4	40	6.9	68	15.6	78	8.6
Recreational solvent inhalation: ever	327	29.1	124	22.8	203	35.7	157	36.0	170	21.9
Marijuana use: daily/regular (> once/month)	588	48.3	316	52.4	272	44.1	255	57.3	333	38.9
Second-hand smoke exposure: almost daily	417	33.2	190	30.6	227	35.8	177	37.7	241	28.4
Body mass index										
<18.5 (underweight)	31	1.8	16	1.8	15	1.7	5	1.0	25	2.5
18.5–24.9 (healthy)	514	42.5	294	50.2	220	34.5	222	50.1	293	34.4
25–29.9 (overweight)	346	25.9	163	24.8	183	26.9	114	24.0	232	27.8
≥ 30 (obesity)	405	29.9	156	23.2	249	36.8	112	24.8	293	35.3
Marine mammal and fish consumption										
< 3 times/week	392	28.9	190	27.6	201	30.2	119	25.8	273	32.2
3–6 times/week	393	29.9	211	33.9	182	25.8	130	28.8	263	31.1
≥ 7 times/week	511	41.2	228	38.5	283	44.0	204	45.4	307	36.7
Fruits/vegetables: ≥ 5 times/day	200	14.9	101	14.5	99	15.3	59	13.0	141	16.9
Vitamin D: sufficient blood level	909	67.9	344	55.1	564	81.1	312	65.9	597	70.1
Respiratory infection during childhood	86	6.8	19	3.3	67	10.3	25	5.6	61	8.0
Active TB in the past	103	7.3	15	2.6	88	12.1	34	6.7	69	7.9
Allergic sensitization to dogs	43	3.1	23	3.4	20	2.8	15	3.0	28	3.2
Allergic sensitization to dust mites	80	5.8	33	5.2	47	6.4	26	5.8	54	5.9
High blood total IgE (≥ 100 kU/L)	347	25.5	132	20.9	215	30.4	132	26.7	215	24.3
Housing crowding (> 1 person per room)	423	32.0	252	37.5	171	26.4	137	30.5	286	33.7
Housing in need of major repairs	251	19.1	111	18.0	140	20.2	91	20.0	160	18.2
Food security										
Food secure	441	32.9	190	29.2	250	36.7	135	29.9	305	36.0
Moderately food insecure	610	48.1	319	51.5	291	44.5	215	48.6	395	47.4
Severely food insecure	245	19.0	119	19.3	126	18.8	103	21.4	142	16.5
Personal income										
< $15,000	533	41.2	342	53.2	191	28.8	176	39.9	356	42.6
$15,000–$24,999	251	19.2	131	20.1	119	18.3	88	18.9	163	19.6
$25,000–$39,999	161	13.0	69	12.5	92	13.6	67	14.8	94	11.1
$40,000–$59,999	154	13.0	49	9.3	105	16.7	60	14.5	94	11.4
≥ $60,000	198	13.6	38	4.9	160	22.5	62	11.9	136	15.3
School level										
Post-secondary	91	5.6	14	1.6	77	9.6	19	3.4	72	7.8
High school completed	292	23.6	160	26.2	132	20.8	103	23.8	189	23.3
Some high school or less	913	70.9	455	72.1	458	69.6	331	72.7	582	68.9
Going on the land: often	550	43.5	276	44.6	274	42.5	193	44.8	357	42.3
Traditional activities: in the last year	1167	89.4	569	89.5	599	89.4	392	87.6	776	91.4

Data are pooled from multiple imputations, except for age and sex, for which there were no missing data. Numbers (*N*) are rounded and unweighted to represent the sample while proportions (%) are weighted to represent Nunavik’s population. No statistical comparisons were performed between groups

In this relatively young population (83% aged 16–54 years old), wheezing was most prevalent (27%), followed by chronic cough (21%) and airway obstruction (17%) (Table 2). The prevalence of wheezing was similar across age groups and sexes. The prevalence of chronic cough significantly increased with age but did not differ between sexes. In contrast, the prevalence of airway obstruction was significantly higher among men than among women but did not vary across age groups.

**Table 2:** Prevalence of wheezing, chronic cough, and airway obstruction; population of Nunavik aged 16 and over, 2017

	Wheezing		Chronic cough		Airway obstruction	
	*N*	% (95% CI)	*N*	% (95% CI)	*N*	% (95% CI)
Total population	342	27.2 (24.4–30.1)	262	20.5 (17.9–23.0)	176	16.8 (14.0–19.6)
16–34 years	156	26.4 (22.3–30.5)	96	13.9 (10.9–16.9)*	93	17.0 (13.2–20.9)
≥ 35 years	186	28.1 (24.2–32.0)	166	27.1 (23.1–31.2)*	83	16.6 (12.5–20.7)
Men	126	28.0 (23.4–32.6)	86	19.2 (15.1–23.3)	79	19.5 (14.8–24.3)^§^
Women	216	26.5 (23.2–29.7)	176	21.8 (18.7–24.8)	97	14.0 (11.1–16.9)^§^

Numbers (*N*) are unweighted to represent the sample while proportions (%) are weighted to represent Nunavik’s population. Airway obstruction was defined as a FEV_1_/FVC ratio below the lower limit of normal (LLN).

*p < 0.05 for the difference between age groups (bilateral Wald chi-square test)

^§^*p* < 0.05 for the difference between sexes (bilateral Wald chi-square test)

Tables 3 to 5 present the results of the multivariate logistic regression models that examined the effect of various characteristics on wheezing, chronic cough, and airway obstruction, for the total population, each age group, and each sex.

**Table 3:** Multivariable logistic models for wheezing, population of Nunavik aged 16 and over, 2017

	Total population (*N* = 1258)	16–34 years old (*N* = 608)	35 years and older (*N* = 650)	Men (*N* = 439)	Women (*N* = 819)
	OR (95% CI)	OR (95% CI)	OR (95% CI)	OR (95% CI)	OR (95% CI)
Sex: men versus women	1.00 (0.73–1.37)	0.99 (0.59–1.63)	1.04 (0.65–1.66)		
Tobacco smoking					
Never	Ref	0.04 (0.00–0.44)*	Ref	Ref	Ref
Former	2.24 (1.13–4.46)*	1.59 (0.73–3.46)	1.35 (0.59–3.08)	2.98 (1.08–8.20)*	1.86 (0.71–4.84)
Current < 15 pack-years	1.80 (1.00–3.23)	Ref^A^	1.15 (0.53–2.51)	1.77 (0.78–4.04)	2.00 (0.83–4.81)
Current ≥ 15 pack-years	3.13 (1.63–5.99)*	3.32 (1.17–9.37)*	2.08 (0.99–4.37)	3.17 (1.26–7.94)*	3.59 (1.37–9.42)*
Recreational solvent: ever, at least once	1.33 (0.93–1.91)	2.62 (1.53–4.51)*	0.72 (0.43–1.18)	1.16 (0.65–2.07)	1.56 (1.01–2.41)*
Marijuana use: daily/regular (> once/month)	1.08 (0.77–1.51)	1.12 (0.67–1.86)	1.05 (0.64–1.70)	0.92 (0.53–1.61)	1.18 (0.80–1.75)
Second-hand smoke exposure: almost daily	1.19 (0.85–1.67)	1.47 (0.87–2.48)	1.15 (0.70–1.88)	1.30 (0.77–2.21)	1.09 (0.72–1.67)
Body mass index (vs. 18.5–24.9)					
<18.5 (underweight)	1.45 (0.51–4.12)	1.48 (0.28–7.86)	2.69 (0.56–12.92)	0.71 (0.05–10.00)	1.78 (0.58–5.50)
25–29.9 (overweight)	1.56 (1.03–2.36)*	1.77 (0.94–3.33)	1.64 (0.87–3.10)	1.81 (0.94–3.49)	1.45 (0.87–2.43)
≥ 30 (obesity)	2.18 (1.46–3.28)*	1.42 (0.71–2.84)	3.09 (1.68–5.68)*	2.36 (1.20–4.64)*	1.93 (1.17–3.20)*
Marine mammals and fish (vs. < 3 times/week)					
3–6 times/week	1.46 (0.97–2.22)	1.76 (0.90–3.47)	1.21 (0.66–2.24)	1.91 (0.90–4.05)	1.25 (0.77–2.03)
≥ 7 times/week	1.78 (1.21–2.63)*	1.76 (0.90–3.43)	1.76 (1.01–3.07)*	2.76 (1.43–5.32)*	1.31 (0.82–2.09)
Fruits/vegetables: ≥ 5 times/day	1.56 (1.03–2.35)*	1.15 (0.60–2.20)	2.54 (1.38–4.70)*	1.20 (0.59–2.44)	1.85 (1.10–3.09)*
Vitamin D: sufficient blood level	NS	0.87 (0.52–1.45)	0.57 (0.32–1.02)	1.23 (0.67–2.26)	1.40 (0.90–2.18)
Respiratory infection during childhood	1.67 (0.96–2.90)	1.07 (0.34–3.33)	1.97 (0.99–3.92)	1.32 (0.50–3.51)	1.96 (0.96–3.97)
Allergic sensitization to dogs	2.60 (1.11–6.08)*	3.59 (0.92–14.07)	1.85 (0.56–6.05)	5.74 (1.13–29.04)*	1.30 (0.42–3.96)
High blood total IgE	NS	0.79 (0.43–1.48)	1.32 (0.79–2.20)	0.89 (0.48–1.67)	1.14 (0.73–1.78)
Housing crowding (> 1 person per room)	0.52 (0.36–0.75)*	0.65 (0.38–1.12)	0.40 (0.23–0.72)*	0.64 (0.36–1.15)	0.42 (0.27–0.65)*
Housing in need of major repairs	1.20 (0.82–1.75)	NS	NS	0.90 (0.48–1.69)	1.64 (1.03–2.61)*
Food security (vs. severely food insecure)					
Food secure	0.44 (0.28–0.69)*	0.46 (0.21–1.00)*	0.27 (0.14–0.51)*	0.45 (0.21–0.95)*	0.39 (0.22–0.67)*
Moderately food insecure	0.52 (0.34–0.79)*	0.64 (0.34–1.22)	0.30 (0.16–0.56)*	0.54 (0.28–1.03)	0.49 (0.29–0.81)*
Personal income (vs. < $15,000)					
$15,000–$24,999	NS	1.22 (0.62–2.38)	1.07 (0.55–2.10)	NS	NS
$25,000–$39,999	NS	2.69 (1.20–6.01)*	0.63 (0.28–1.38)	NS	NS
$40,000–$59,999	NS	0.74 (0.24–2.26)	0.76 (0.35–1.67)	NS	NS
≥ $60,000	NS	3.18 (1.02–9.85)*	1.17 (0.60–2.30)	NS	NS
School attainment (vs. high school not completed)					
Post-secondary	NS	NS	NS	NS	NS
High school completed	NS	NS	NS	NS	NS
Traditional activities: in the last year	NS	1.34 (0.58–3.10)	1.20 (0.55–2.59)	0.99 (0.46–2.11)	1.72 (0.81–3.67)
Going on the land: often	NS	1.19 (0.72–1.97)	1.02 (0.65–1.59)	NS	NS

For each group, the model includes age (16–24, 25–34, 35–49, 50–59, ≥ 60) and all variables that are presented in the column, except if indicated as “NS” for “not selected”

*p < 0.05

^A^The reference category is different for young adults because the number of cases among never smokers was too small to yield reliable estimates

**Table 4:** Multivariable logistic models for chronic cough, population of Nunavik aged 16 and over, 2017

	Total population (*N* = 1257)	16–34 years old (*N* = 609)	35 years and older (*N* = 648)	Men (*N* = 437)	Women (*N* = 820)
	OR (95% CI)	OR (95% CI)	OR (95% CI)	OR (95% CI)	OR (95% CI)
Sex: men versus women	0.70 (0.48–1.04)	0.49 (0.27–0.89)*	0.97 (0.58–1.63)		
Tobacco smoking					
Never	ref	0.18 (0.03–0.94)*	ref	ref	ref
Former	0.97 (0.41–2.34)		0.87 (0.33–2.30)	1.05 (0.26–4.21)	0.97 (0.30–3.09)
Current, < 15 pack-years	1.74 (0.86–3.52)	Ref^A^	1.12 (0.48–2.61)	2.02 (0.70–5.82)	1.62 (0.64–4.13)
Current, ≥ 15 pack-years	3.39 (1.59–7.20)*	1.27 (0.39–4.15)	2.66 (1.15–6.12)*	2.98 (1.00–8.88)*	3.87 (1.40–10.74)*
Electronic cigarette: in past year	1.25 (0.76–2.05)	NS	NS	0.82 (0.39–1.70)	2.11 (1.16–3.85)*
Recreational solvent: ever, at least once	0.99 (0.64–1.51)	1.66 (0.87–3.17)	0.71 (0.41–1.22)	NS	NS
Marijuana use: daily/regular (> once/month)	1.25 (0.85–1.85)	0.99 (0.52–1.86)	1.42 (0.85–2.38)	0.94 (0.49–1.83)	1.52 (0.97–2.38)
Second-hand smoke exposure: almost daily	1.05 (0.71–1.56)	1.04 (0.53–2.04)	1.10 (0.63–1.92)	1.12 (0.57–2.20)	0.96 (0.61–1.50)
Body mass index (vs. 18.5–24.9)					
< 18.5 (underweight)	2.19 (0.72–6.60)	0.35 (0.06–1.90)	0.41 (0.07–2.51)	1.40 (0.08–25.61)	2.40 (0.74–7.78)
25–29.9 (overweight)	1.23 (0.76–1.99)	0.49 (0.08–2.96)	0.47 (0.07–2.94)	1.40 (0.64–3.05)	1.13 (0.64–1.99)
≥ 30 (obesity)	1.42 (0.86–2.33)	0.44 (0.07–2.72)	0.66 (0.10–4.15)	1.28 (0.51–3.17)	1.46 (0.83–2.55)
Marine mammals and fish (vs. < 3 times/week)					
3–6 times/week	1.32 (0.80–2.20)	1.36 (0.60–3.05)	1.52 (0.76–3.04)	1.71 (0.70–4.20)	1.21 (0.68–2.16)
≥ 7 times/week	2.19 (1.39–3.44)*	3.89 (1.90–7.99)*	1.80 (0.97–3.35)	3.25 (1.46–7.22)*	1.82 (1.06–3.11)*
Fruits/vegetables: ≥ 5 times/day	1.22 (0.76–1.97)	NS	NS	1.43 (0.63–3.27)	1.09 (0.61–1.95)
Respiratory infection during childhood	2.12 (1.07–4.18)*	2.8 (0.77–10.11)	2.37 (1.04–5.38)*	2.33 (0.70–7.77)	2.33 (1.09–4.96)*
Active TB in the past	1.82 (0.88–3.75)	0.62 (0.06–6.36)	2.14 (0.93–4.94)	1.54 (0.46–5.13)	2.36 (1.00–5.55)*
Allergic sensitization to dogs	NS	1.68 (0.36–7.82)	0.12 (0.01–1.03)	NS	NS
Housing crowding (> 1 person per room)	1.50 (1.01–2.21)*	2.15 (1.18–3.90)*	1.25 (0.71–2.22)	1.72 (0.86–3.45)	1.42 (0.91–2.22)
Housing in need of major repairs	1.72 (1.13–2.61)*	2.50 (1.25–5.02)*	1.54 (0.87–2.71)	2.18 (1.09–4.38)*	1.46 (0.87–2.43)
Food security (vs. severely food insecure)					
Food secure	0.25 (0.14–0.43)*	0.23 (0.09–0.64)*	0.24 (0.11–0.50)*	0.19 (0.07–0.53)*	0.27 (0.14–0.51)*
Moderately food insecure	0.47 (0.30–0.73)*	0.64 (0.31–1.32)	0.37 (0.19–0.70)*	0.49 (0.23–1.02)	0.45 (0.26–0.76)*
Personal income (vs. < $15,000)					
$15,000–$24,999	2.55 (1.53–4.27)*	2.40 (1.11–5.16)*	2.84 (1.32–6.12)*	2.77 (1.14–6.74)*	2.47 (1.33–4.58)*
$25,000–$39,999	1.80 (0.95–3.42)	1.86 (0.64–5.44)	1.84 (0.78–4.32)	2.23 (0.84–5.88)	1.24 (0.55–2.79)
$40,000–$59,999	1.59 (0.79–3.21)	1.01 (0.27–3.78)	1.68 (0.70–4.03)	1.84 (0.62–5.46)	1.27 (0.54–3.00)
≥ $60,000	2.18 (1.05–4.50)*	9.84 (2.60–37.21)*	1.66 (0.73–3.75)	2.26 (0.55–9.22)	1.98 (0.92–4.24)
School attainment (vs. high school not completed)					
Post-secondary	1.11 (0.56–2.20)	1.05 (0.15–7.61)	1.03 (0.49–2.17)	NS	NS
High school completed	0.73 (0.45–1.18)	0.94 (0.46–1.91)	0.61 (0.31–1.18)	NS	NS
Traditional activities: in the last year	0.82 (0.47–1.43)	1.03 (0.42–2.53)	0.70 (0.32–1.52)	0.79 (0.32–1.93)	0.89 (0.44–1.80)
Going on the land: often	0.61 (0.41–0.88)*	0.55 (0.30–1.02)	0.62 (0.38–1.02)	0.50 (0.26–0.94)*	0.73 (0.47–1.14)

For each group, the model includes age (16–24, 25–34, 35–49, 50–59, ≥ 60) and all variables that are presented in the column, except if indicated as “NS” for “not selected”

*p < 0.05

^A^The reference category is different for young adults because the number of cases among never and former smokers was too small to yield reliable estimates. The two categories were combined because no never smoker had chronic cough

**Table 5:** Multivariable logistic models for airway obstruction, population of Nunavik aged 16 and over, 2017

	Total population (*N* = 1088)	16–34 years old (*N* = 535)	35 years and older (*N* = 553)	Men (*N* = 376)	Women (*N* = 712)
	OR (95% CI)	OR (95% CI)	OR (95% CI)	OR (95% CI)	OR (95% CI)
Sex: men versus women	1.30 (0.85–2.00)	1.25 (0.70–2.25)	1.29 (0.70–2.39)		
Tobacco smoking					
Never	Ref	0.84 (0.30–2.38)	Ref	Ref	Ref
Former	1.40 (0.52–3.72)	0.98 (0.27–3.53)	1.62 (0.46–5.73)	1.77 (0.57–5.49)	1.55 (0.25–9.53)
Current, < 15 pack-years	1.12 (0.52–2.41)	Ref^A^	0.89 (0.23–3.39)	0.81 (0.31–2.13)	3.55 (0.85–14.79)
Current, ≥ 15 pack-years	2.86 (1.18–6.95)*	1.58 (0.44–5.74)	3.17 (0.95–10.57)	2.23 (0.75–6.64)	9.67 (2.15–43.51)*
Marijuana use: daily/regular (> once/month)	1.54 (0.97–2.43)	2.22 (1.19–4.14)*	1.30 (0.65–2.62)	1.32 (0.67–2.58)	1.84 (1.08–3.13)*
Second-hand smoke exposure: almost daily	1.01 (0.64–1.58)	0.70 (0.34–1.41)	1.63 (0.84–3.15)	NS	NS
Body mass index (vs. 18.5–24.9)					
< 18.5 (underweight)	3.84 (1.12–13.19)*	10.61 (1.89–59.42)*	2.03 (0.35–11.75)	2.48 (0.14–42.67)	4.11 (1.11–15.17)*
25–29.9 (overweight)	1.05 (0.64–1.74)	1.15 (0.55–2.39)	0.87 (0.39–1.91)	1.08 (0.52–2.23)	0.95 (0.51–1.76)
≥ 30 (obesity)	0.63 (0.32–1.21)	0.45 (0.19–1.09)	0.65 (0.27–1.54)	0.60 (0.22–1.59)	0.62 (0.30–1.29)
Marine mammals and fish (vs. < 3 times/week)					
3–6 times/week	NS	NS	NS	0.85 (0.38–1.92)	2.13 (1.09–4.16)*
≥ 7 times/week	NS	NS	NS	0.77 (0.37–1.60)	1.06 (0.53–2.13)
Vitamin D: sufficient blood level	NS	1.14 (0.63–2.05)	0.60 (0.27–1.33)	NS	NS
Respiratory infection during childhood	1.82 (0.76–4.34)	NS	NS	NS	NS
Active TB in the past	NS	0.17 (0.02–1.91)	1.04 (0.33–3.23)	NS	NS
Allergic sensitization to dogs	1.92 (0.69–5.36)	1.13 (0.25–5.01)	2.76 (0.73–10.42)	1.69 (0.40–7.22)	1.89 (0.55–6.54)
Housing crowding (> 1 person per room)	NS	1.15 (0.60–2.20)	0.59 (0.27–1.29)	NS	NS
Food security (vs. severely food insecure)					
Food secure	NS	1.07 (0.42–2.69)	1.23 (0.58–2.61)	NS	NS
Moderately food insecure	NS	1.01 (0.44–2.31)	0.57 (0.25–1.28)	NS	NS
School attainment (vs. high school not completed)					
Post-secondary	0.52 (0.02–1.37)	1.37 (0.22–8.47)	0.41 (0.13–1.32)	NS	NS
High school completed	0.96 (0.56–1.66)	0.97 (0.50–1.92)	0.90 (0.41–2.02)	NS	NS
Traditional activities: in the last year	NS	1.51 (0.57–3.98)	0.77 (0.34–1.74)	NS	NS

For each group, the model includes age (16–24, 25–34, 35–49, 50–59, ≥ 60) and all variables that are presented in the column, except if indicated as “NS” for “not selected.” Airway obstruction was defined as a FEV_1_/FVC ratio below the lower limit of normal (LLN). Sensitivity analysis with the fixed ratio (0.7) is presented in Supplementary material

^A^The reference category is different for young adults because the number of cases among never smokers was too small to yield reliable estimates

*p < 0.05

### Characteristics associated with wheezing

Tobacco smoking was the main characteristic associated with wheezing (Table 3). Odds of wheezing were three times higher among people who had smoked at least 15 pack-years of cigarettes, compared to never smokers. Wheezing was also more frequent among other current smokers (almost significant) and former smokers. Odds of wheezing were increased twofold by obesity and by allergic sensitization to dogs. Odds were higher with fruit/vegetable consumption (≥ 5 times/day) and fish/marine mammal consumption (≥ 7 times/week) and tended to be higher with hospitalization for respiratory infection during childhood. Odds were lower among those who lived in crowded housing.

Stratified analysis revealed similar results concerning tobacco smoking but unveiled some differences between age groups and sexes. Among young adults, having ever used solvents for recreational purposes was a risk factor of wheezing. Obesity, fish/marine mammal consumption, fruit/vegetable consumption, and housing crowding were significant among adults (≥ 35 years) only. Allergic sensitization to dogs and fish/marine mammal consumption were risk factors among men only. Recreational use of solvent, housing in need of major repairs, and fruit/vegetable consumption were risk factors among women only.

### Characteristics associated with chronic cough

Tobacco smoking was the strongest risk factor of chronic cough: odds of chronic cough were three times higher among current smokers who had smoked at least 15 pack-years (Table 4). Both crowding and housing in need of repairs independently increased the risk of chronic cough. Other risk factors were hospitalization for respiratory infection during childhood and fish/marine mammal consumption (≥ 7 times/week).

Tobacco smoking was a risk factor of chronic cough in both age groups. In fact, none of the 49 young never smokers reported chronic cough. Some associations were significant only among young adults: housing crowding, housing in need of major repairs, and fish/marine mammal consumption. Hospitalization for respiratory infection during childhood increased the odds of chronic cough in both age groups but was significant only among adults (≥ 35 years). Fish/marine mammal consumption was a stronger risk factor of chronic cough in men than in women. Conversely, electronic cigarette use and prior active TB were risk factors among women only.

### Characteristics associated with airway obstruction

Major characteristics associated with airway obstruction (Table 5) were being underweight and current smoking (≥ 15 pack-years). Smoking less than 15 pack-years was not associated with airway obstruction. In adults (≥ 35 years), airway obstruction tended to be associated with tobacco smoking (≥ 15 pack-years, OR = 3.17 [0.95–10.57]) but not with regular marijuana use. The opposite is observed in young adults: airway obstruction was associated with regular marijuana use, but not with tobacco smoking. Of note, few young adults had smoked 15 pack-years at the time of the survey even if the majority were smokers (Table 1). Among women, both current smoking and marijuana use were independent risk factors. Confidence intervals were large because only 61 women never smoked, and only three of them had obstruction. No consistent pattern was found between airway obstruction and fish/marine mammal consumption. There was no significant association among men. Defining airway obstruction with the fixed ratio instead of LLN did not result in important changes in the prevalence of airway obstruction or its associated characteristics (Supplementary material C, Tables C5 to C7).

### Social determinants

As explained in the Methods section, social determinants presumably influence respiratory health though the mediation of other factors. Since a variable should not be adjusted for its mediators, we reported here the results from minimally adjusted models (age and sex only), presented in Supplementary material C, Tables C2 to C4. Being food secure was strongly protective against wheezing (Table C2). Severe insecurity was worse than moderate insecurity, indicating a graded relationship. Higher income (≥ $60,000) tended to be associated with lower odds of wheezing (OR = 1.59 [1.00–2.55]), but the relation between income and wheezing was inconsistent and did not exhibit a social gradient. Food security, participating in traditional activities, and often going on the land were protective against chronic cough (Table C3), while completion of high school was almost significant (OR = 0.67 [0.44–1.01]). Finally, post-secondary education was protective against airway obstruction (Table C4).

### Summary of results

**Fig. 2:**
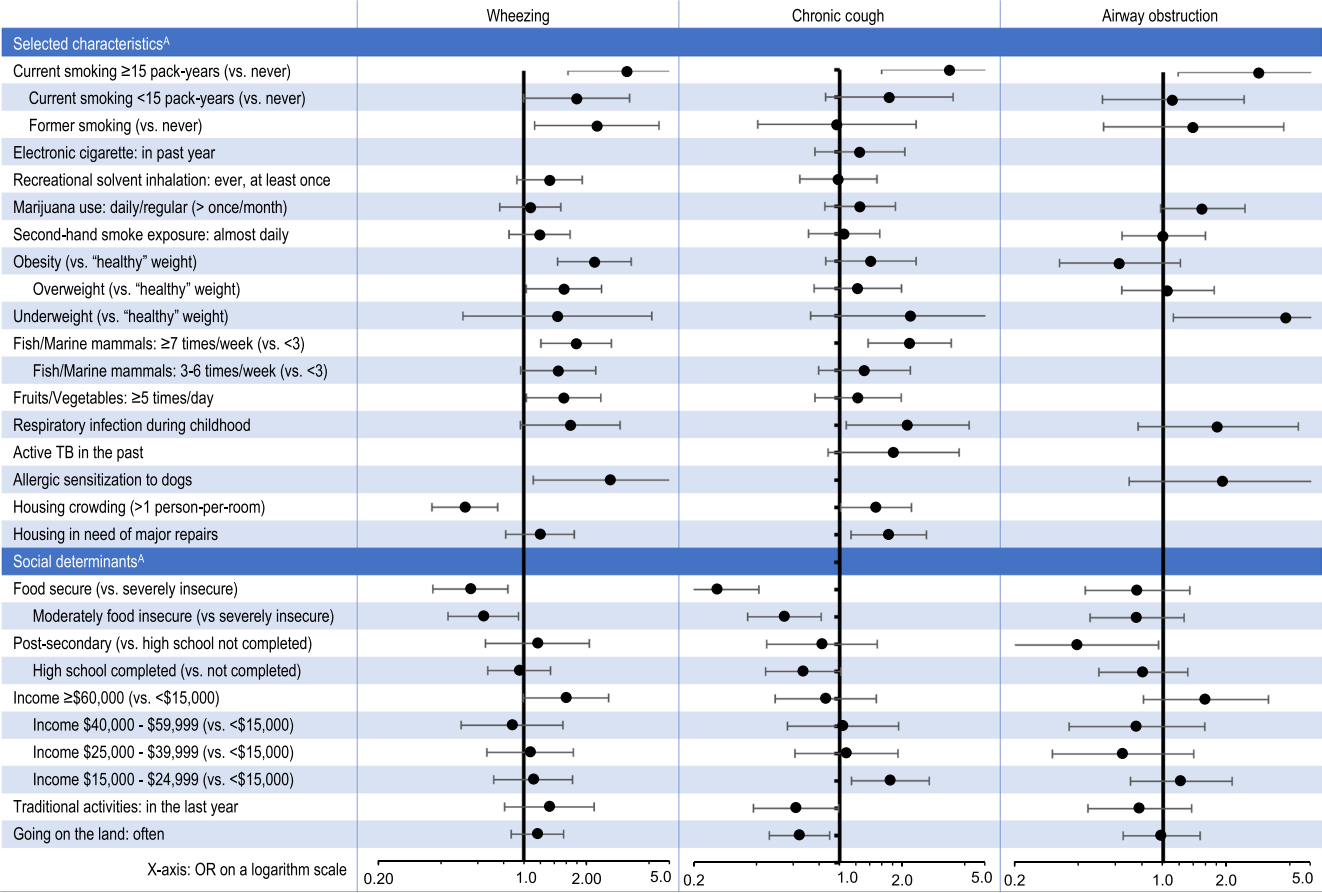
Forest plots of odds ratios (OR and 95% CI) for wheezing, chronic cough, and airway obstruction; population of Nunavik aged 16 and over, 2017. ^A^ For social determinants, odds ratios are adjusted for age and sex, as explained in the text (results in Tables C2-C5 in Supplementary Material). Odds ratios for selected characteristics are fully adjusted (results in Tables 3 to 5)

Forest plots (Fig. 2) summarize the results in the total population. Please note that for social determinants, odds ratios correspond to age/sex-adjusted models presented in Tables C2 to C4 in Supplementary material C.

## Discussion

Wheezing, chronic cough, and airway obstruction are relatively frequent respiratory health indicators in Nunavik, affecting a sixth to a quarter of the population. The prevalence of wheezing in Nunavik (27%) is similar to the prevalence noted in CanCOLD, a multicentric study of Canadians aged 40 years and older (29%^1^) (Tan et al., 2015). It is also similar to values in “high-prevalence” countries according to the World Health Survey (To et al., 2012). Of note, these countries had a much higher prevalence of physician-diagnosed asthma (around 15–22%) than the one previously reported in Nunavik (4%) (Robert et al., 2020). The prevalence of wheezing among adults in the present survey is higher than among 3–5-year-old children from Nunavut in 2007–2008 (23%) (Kovesi et al., 2011), and much higher than the prevalence among 5–14-year-old children from Nunavik in 2004 (3.4%) (Lajoie et al., 2004). In contrast, diagnosed asthma remains relatively infrequent in adults (Supplementary material A). This suggests some underdiagnosis of asthma, which could be explained by the fact that up until 2019, spirometry testing was available only for the residents of Hudson Coast communities for one week, twice annually. The prevalence of chronic cough is twofold higher in Nunavik (27% among adults aged ≥ 35 years old) than among CanCOLD participants (13%^2^) (Tan et al., 2015). It is comparable to that of other countries with high prevalence, i.e., over 20% (Song et al., 2015). Recent Canadian population health surveys did not document the prevalence of wheezing and chronic cough, but CHMS estimated the prevalence of airway obstruction to be 17% in Canada (Evans et al., 2014), and 12% after adjustment for the younger age distribution in Nunavik (Robert et al., 2020). Hence these indicators seem more prevalent in Nunavik than in the rest of Canada, or similar to countries with high prevalence, which is consistent with higher hospitalization and mortality rates from respiratory causes observed in Nunavik (NRBHSS, 2015). These data show the need for increased access to primary care and spirometry in Nunavik.

Many characteristics were associated with respiratory health indicators, with some general tendencies across indicators, age groups, and sexes. Risk factors included tobacco smoking, obesity, underweight, respiratory infections during childhood, allergic sensitization to dogs, and housing in need of major repairs, while protective factors included food security, school attainment, going on the land, and participating in traditional activities. Housing crowding was associated with higher odds of chronic cough but lower odds of wheezing. The strongest and most consistent associations were observed with current smoking (≥ 15 pack-years). The benefits of smoking cessation are apparent: having smoked less than 15 pack-years did not increase the risk of chronic cough and airway obstruction, and former smokers were not more at risk of airway obstruction than never smokers. While evidence is still mixed and insufficient to conclude about an association between marijuana use and airway obstruction, it is clearly associated with wheezing and cough according to a recent meta-analysis (Ghasemiesfe et al., 2018). The design of this survey does not permit providing epidemiologic evidence of causal effect related to marijuana use. Nevertheless, it shows that current tobacco smoking and regular marijuana use were both highly prevalent in Nunavik (78.9% and 48.3%, respectively), a situation that has roots in the social determinants of health.

Ever using solvents for recreational purposes was associated with wheezing and airway obstruction among young people and women. Although we lacked data about the frequency and duration of exposure, solvent abuse was included as an exploratory variable because solvents are irritating for the respiratory tract and are thought to trigger wheezing or dyspnea following inhalation (Brouette & Anton, 2001). Recreational users could inhale much higher concentrations than occupational exposure limits (Meadows & Verghese, 1996). While neuropsychological, cardiac, and psychosocial effects are recognized (Baydala, 2010), effects on lung function are largely unknown; we found only two series of about 30 cases that reported a restrictive pattern among some users (Büker et al., 2011; Pogoy & de Guia, 2005).

Food security was consistently protective against respiratory symptoms, exhibiting one of the strongest and most consistent associations. It is an indicator of socioeconomic status, a known predictor of respiratory diseases (GOLD, 2019; Uphoff et al., 2015). Its effect remained significant in fully adjusted models. Potential mediators include nutritional factors (like vitamin and antioxidant intakes) and psychosocial stress (Eisner et al., 2010; GINA, 2018; Yonas et al., 2012). The majority of Nunavik population experienced moderate or severe food insecurity (67.1%), versus 8.8% of Canadian households in 2017 (Statistics Canada, 2020), highlighting the pervasive food insecurity crisis in Northern Canada. Even if more studies are needed to confirm its impact on respiratory health, food insecurity is associated with many other consequences on self-rated health, mental health, underweight but also overweight, diabetes, and cardiovascular diseases (Council of Canadian Academies, 2014).

Higher school attainment seemed protective against chronic cough and airway obstruction, with large confidence intervals that reflect the small number of participants in higher categories of school attainment, speaking for social inequities faced by Indigenous communities. Personal income had a variable effect across its categories, without the usual social gradient, perhaps because the self-reported estimation of personal income before taxes does not consider the family’s income and size. Food insecurity might be a better indicator of financial stress than education or income in Inuit communities, at least for respiratory health, as observed among Indigenous communities in Australia (Cunningham, 2010). Cultural characteristics were protective against chronic cough, especially among adults (≥ 35 years). They are considered protective for Inuit health and could influence lifestyle (Reading & Wien, 2009). Findings could also indicate that respiratory conditions restrict people’s capacity to go on the land.

Poor housing quality and housing crowding were both associated with an increased prevalence of chronic cough, while crowding was associated with a lower prevalence of wheezing. The need of major repairs is an indicator of problems with heating, electrical, plumbing, or house structure (Statistics Canada, 2017). Possible consequences are reduced air quality, decreased indoor temperature, and mould growth, which affect respiratory health (Institute of Medicine of the National Academies, 2000). Housing crowding can also impact respiratory health, for example, by increasing the risk of respiratory infections or exposure to second-hand smoke (Basnayake et al., 2017). Based on these mechanisms, the reduced risk of wheezing was unexpected. A recent prospective study in Nunavik and Nunavut found a significant reduction in asthma-like symptoms after rehousing and reduction of crowding (Riva et al., 2019). Alternatively, the “hygiene theory” postulates that exposure to germs prevents allergic sensitization and asthma (GINA, 2018). Kovesi (2012) cited this theory to explain the low frequency of asthma among Inuit children, despite high rates of respiratory infections. Allergic sensitization is indeed less frequent among Inuit than in other populations (Hemmelgarn & Ernst, 1997; Porsbjerg et al., 2002). The prevalence of allergic sensitization to dogs (3.1%) was much lower than among American adults aged 20–69 years measured in NHANES 2005–2006 (9–14% depending on the age group, according to Salo et al., 2014). Similarly, allergic sensitization to dust mites was much less prevalent in our study (5.8%) than among Canadian adults from six cities (33–43% depending on the age group and the *Dermatophagoides* species, according to Chan-Yeung et al., 2010). Yet, some people exhibit features of allergic asthma: sensitization to dogs was significantly associated with wheezing, especially among men. Additional research is needed to clarify which type of asthma is most prevalent in Nunavik and its associated risk factors.

Our findings also support an adverse effect of severe respiratory infections during childhood on respiratory health. Similarly, childhood hospitalization for respiratory illness was associated with COPD in CanCOLD and BOLD studies (Hooper et al., 2012; Tan et al., 2015). It is noteworthy that the medical file review included only hospitalizations in Kuujjuaq, Puvirnituq, or a southern hospital. Prolonged stays (> 24 h) in community health centres for respiratory infections are much more frequent among Nunavik children—57% according to Dallaire et al. (2006)—but were not counted as hospitalization during the file review. This could have reduced the strength of the association. Prior active TB was generally not associated with respiratory indicators, even if TB is a likely cause of lung function decline and irreversible airway obstruction (Eisner et al., 2010). This lack of association might be explained by a higher proportion of missing data regarding active TB in the medical files or by the small number of cases.

The relation between respiratory symptoms and fish/marine mammal consumption was unexpected, since these foods were included for their potential benefits. No study found a deleterious effect of fish consumption in a meta-analysis; pooled effects were protective among children and not significant among adults (Yang et al., 2013). We combined fish and marine mammals since they share common beneficial nutrients such as n-3 PUFA and antioxidants (Baines et al., 2015). We examined their separate effects in a post hoc analysis presented in the Supplementary material (Tables C8–C12). The odds ratio for each food group was closer to the null value and no longer significant in the whole population, and findings were similar to primary analyses for chronic cough among subgroups. Further analyses are required using other variables such as the status of specific nutrients, or exposure to cold while fishing. In any case, we believe these findings do not question the promotion of these culturally important foods with established benefits for health and food security.

Few characteristics were significantly associated with airway obstruction among Nunavimmiut, in contrast to findings of larger population-based studies in Canada, the United States, and elsewhere (Halldin et al., 2015; Hooper et al., 2012; Tan et al., 2015). Among risk factors of obstruction in the latter studies, three characteristics were also significant in our analyses (smoking, underweight, education level); one followed the same trend (obesity); one was associated with other indicators (childhood hospitalization for respiratory illness); and two were generally not associated (prior TB disease, second-hand smoke). The smaller sample size of the current survey could have contributed to these differences. The association between underweight and airway obstruction is well recognized, but its direction and mechanism remain unclear (Vanfleteren et al., 2016).

### Strengths and limitations

This study provides a holistic overview of personal, lifestyle, environmental, social, and cultural characteristics associated with respiratory health in Nunavik, based on a relatively large sample, comprising approximately one tenth of the Nunavik population of this age. A thousand spirometry tests were conducted despite the challenges of performing such tests in isolated communities. Three prevalent respiratory indicators represented complementary aspects of respiratory health, including both objective and subjective measures. Few studies had previously described the prevalence of spirometry-measured airway obstruction (or COPD) among young adults, and its associated characteristics.

The low participation rate among sampled individuals might have introduced a selection bias that could not be fully corrected with weighting, but it could be mitigated by the high participation rate among reached individuals (80%). The cross-sectional design is an inherent limit of this population-based survey. Associations could be missed or underestimated because exposures (e.g., housing or food security) may have changed through the years, or because some individuals may have modified their behaviours after learning about their disease. Nevertheless, cross-sectional studies are still considered useful for progressive diseases like respiratory diseases, and for populations that otherwise would not be included in longitudinal studies (Kogevinas & Chatzi, 2015). Most exposures were assessed by questionnaires, which is influenced by participants’ assessment, recall and desirability bias, and by the translation in Inuktitut. Notably, the prevalence of wheezing may have been overestimated because some participants could have been experiencing “laboured breathing” without wheezing. Many participants missed at least one data item, a loss of information that was overcome by multiple imputation. Since many associations were examined, some could be found by chance, so we selected variables based on literature reviews and focused on consistent trends across indicators and groups. Some potential factors were not available, such as genetics and familial history, occupational exposure, and lung development during pregnancy and childhood. Finally, interpretation of prevalence results should consider that wheezing and airway obstruction are not intended to represent the exact prevalence of asthma and COPD, because their diagnosis depends on an individualized evaluation. The prevalence of airway obstruction can overestimate the prevalence of COPD because a bronchodilator is required for the diagnosis; comparisons with CHMS and NHANES III normal values are nevertheless possible because a bronchodilator was also not used in these surveys (Hankinson et al., 1999).

## Conclusion

The prevalence of wheezing, chronic cough, and spirometry-measured airway obstruction confirms the importance of respiratory health in Nunavik. While tobacco smoking is a major determinant of respiratory health, our findings also underscore that efforts must not only focus on smoking prevention, but also on eliminating food insecurity, improving housing availability and quality, and promoting Inuit culture. The protective effect of food security seemed as strong as the deleterious effect of smoking, and food insecurity was almost as prevalent as daily tobacco use. Cultural continuity was already considered vital for well-being and mental health, but the survey findings highlight their potential indirect contribution to physical health. These findings can inform communities as well as regional, provincial, and federal governments in formulating policies that will improve respiratory health by targeting smoking and social determinants, with several co-benefits on physical and mental health. We believe that future research about respiratory health in Nunavik should focus on interventions led and developed by the communities to address the aforementioned determinants. Finally, future research should also target children in Nunavik whose lung health is at risk from early life exposures but who could not be included in the present survey.

## Contributions to knowledge

What does this study add to existing knowledge?

- Respiratory symptoms and airway obstruction are relatively frequent in Nunavik, especially among young adults, partly because of smoking, but presumably also because of socioeconomic conditions.
- Respiratory conditions prevent many people from going on the land and participating in traditional activities.
- Food insecurity, cultural continuity and solvent inhalation are potential determinants of respiratory health that warrant more research in the Inuit context. The protective effect of food security seemed as strong as the deleterious effect of smoking, and food insecurity was almost as prevalent as daily tobacco use.

What are the key implications for public health interventions, practice or policy?

- To some extent, preventing respiratory diseases mainly implies preventing tobacco use, but it also requires downstream action to improve life conditions that influence smoking initiation and cessation.
- Findings can be used in advocacy efforts toward structural policy actions to improve income, food security and housing conditions; respiratory health is only one of several benefits.
- Results can be used in need assessment: lack of access to primary care for respiratory diseases might be considered among other unmet healthcare needs.

### Supplementary Information

ESM 1 (DOCX 87 kb)

## Data Availability

The survey data are owned by Inuit and can be accessed through a request made to *Qanuilirpitaa?* 2017 DMC (nunavikhealthsurvey@ssss.gouv.qc.ca).

## Footnotes

1 Prevalence in the sample; weighted estimate for the Canadian population is not available.

2 Prevalence in the sample; weighted estimate for the Canadian population is not available.
