# Supplementary material for: Respiratory health and its determinants among Nunavimmiut: results from the Qanuilirpitaa? 2017 Nunavik Health Survey
Source: Can J Public Health. 2023 Jan 9;115(Suppl 1):136–51. doi: 10.17269/s41997-022-00722-9 (PMC10830964; doi:10.17269/s41997-022-00722-9)
Supplement: Supplementary file 1 — (DOCX 87 kb) [file 41997_2022_722_MOESM1_ESM.docx]

## Supplementary Materials A, B and C

**Respiratory Health and its Determinants among *Nunavimmiut*: Results from the *Qanuilirpitaa*? 2017 Nunavik Health Survey**

**Authors:**

Philippe Robert^1,2,3^, Benoît Lévesque^1,2,3^, Jean Bourbeau^4,5^, Faiz Ahmad Khan^4,5,6^, Louis-Philippe Boulet^7,8^, Marc-André Dubé^1^, Jean-François Proulx^9^, Pierre Ayotte^1,2,3^

**Author Affiliations:**

^1^ Institut national de santé publique du Québec, Quebec City, QC, Canada

^2^ Département de médecine sociale et préventive, Université Laval, Quebec City, QC, Canada

^3^ Axe Santé des populations et Pratiques optimales en santé, Centre de recherche du CHU de Québec, Quebec City, QC, Canada

^4^ Respiratory Epidemiology and Clinical Research Unit, Department of Medicine, McGill University, Montreal, QC, Canada

^5^, Research Institute, McGill University Health Centre, Montreal Chest Institute, Montreal, QC, Canada

^6^ McGill International TB Centre, Montreal, QC, Canada

^7^ Institut universitaire de cardiologie et de pneumologie de Québec, Quebec City, QC, Canada

^8^ Département de médecine, Université Laval, Quebec City, QC, Canada

^9^ Department of Public Health, Nunavik Regional Board of Health and Social Services, Kuujjuaq, QC, Canada

**Corresponding Author:** Pierre Ayotte, pierre.ayotte@inspq.qc.ca, (418) 650-5115 #4654

Supplementary Material A:

Descriptive Data about Respiratory Health in Nunavik

This study began by a descriptive portrait of respiratory health in Nunavik, presented in a public health report published by the Nunavik Regional Board of Health and Social Services. For convenience, we reproduce here the main descriptive results. Interested readers are referred to the report for a complete description of the methodology and a discussion of the results. We briefly present the variables’ definitions that were not already presented in the article. Lung function was classified in seven mutually exclusive categories, based on GOLD’s classification of airflow limitation severity and the presence of a restrictive syndrome. To identify restrictive syndrome, a pneumologist examined all spirometry curves with FVC lower than 80% of the predicted value: a curve was classified as restrictive if it was suggestive of restriction and if the FEV_1/_FVC ratio was above 0.7; or as mixed if it was suggestive of obstruction and if the FEV_1_/FVC ratio was below 0.7.

Concerning symptoms, “at least one symptom” was an indicator of the burden of respiratory symptoms, defined as a cough (usual but not necessarily chronic) and/or sputum (usual but not necessarily chronic) and/or wheezing (in the past 12 months). Chronic sputum was « bringing up mucus on most days for three months each year, if the person usually brings up mucus from his chest when she does not have a cold ». Breathlessness corresponded to “walking slower than people of the same age on the level because of breathlessness or having to stop for breath when walking at its own pace on the level”. It corresponded to the second stage of the Modified Medical Research Council scale. Chronic bronchitis was defined as “having cough with sputum for at least three months of the year during the past two consecutive years”.

Medical files (outpatient visits, emergency visits and hospitalizations) were reviewed by trained research nurses from their beginning in search of physician diagnoses of asthma, COPD (including emphysema and chronic bronchitis), tuberculosis (latent or active/pulmonary) and hospitalization for respiratory infection during childhood (before the age of 5 years). Physician diagnoses are as written by a doctor in the medical file. We did not verify if asthma and COPD diagnosis were supported by spirometry. Lung cancer diagnosis was searched since 2004. All medications currently prescribed were noted.

Table A1. Lung function in the population of Nunavik aged 16 and over, 2017

|  | % of population | 95%CI | |
| --- | --- | --- | --- |
|  |  | Min | Max |
| Normal | 83.4 | 80.5 | 85.9 |
| Mild obstruction (GOLD 1) | 8.9 | 6.9 | 11.4 |
| Moderate obstruction (GOLD 2) | 5.0 | 3.7 | 6.7 |
| Severe obstruction (GOLD 3) | n.p. | - | - |
| Very severe obstruction (GOLD 4) | n.p. | - | - |
| Mixed syndrome (obstructive and restrictive) | 0.6 | 0.2 | 1.6 |
| Restrictive syndrome | 1.5 | 0.9 | 2.5 |

n.p. Not presented because fewer than 5 participants.

Table A2: Prevalence of airway obstruction in the population of Nunavik aged 16 and over, 2017

|  | Airflow obstruction^A^ | | | Moderate-to-severe airway obstruction^B^ | | |
| --- | --- | --- | --- | --- | --- | --- |
|  | % | 95%CI | | % | 95%CI | |
|  |  | Min | Max |  | Min | Max |
| Total | 16.6 | 14.1 | 19.5 | 6.2 | 4.7 | 8.2 |
| Gender |  |  |  |  |  |  |
| Men | 19.5* | 15.3 | 24.6 | 7.3 | 4.9 | 10.9 |
| Women | 13.6* | 11.1 | 16.5 | 5.1 | 3.7 | 7.1 |
| Age |  |  |  |  |  |  |
| 16-34 | 16.6 | 13.2 | 20.6 | 3.3* | 1.8 | 5.7 |
| 35-49 | 15.3 | 10.0 | 22.8 | 5.4* | 2.9 | 9.9 |
| 50-59 | 20.6 | 14.4 | 28.6 | 15.5* | 9.9 | 23.4 |
| 60-69 | 14.7 | 8.4 | 24.7 | 12.1* | 6.4 | 21.4 |
| 70 + | n.p. | - | - | n.p. | - | - |

*p<0.05 according to a global chi-squared test.

n.p. Not presented because fewer than 5 participants.

^A^ Defined by lower limit of normal.

^B^ Defined by GOLD’s criteria, i.e., the fixed ratio and GOLD’s classification of airflow limitation severity.

Table A3. Prevalence of respiratory symptoms in the population of Nunavik aged 16 and over, 2017

|  | At least one symptom^A^ | | | Wheezing | | | Chronic Sputum | | |
| --- | --- | --- | --- | --- | --- | --- | --- | --- | --- |
|  | % | 95%CI | | % | 95%CI | | % | 95%CI | |
|  |  | Min | Max |  | Min | Max |  | Min | Max |
| Total | 59.0 | 56.0 | 61.9 | 27.1 | 24.3 | 30.0 | 22.5 | 20.0 | 25.2 |
| Sex |  |  |  |  |  |  |  |  |  |
| Men | 57.0 | 53.6 | 60.4 | 28 | 23.6 | 32.8 | 24.4 | 20.4 | 28.9 |
| Women | 59.0 | 56.0 | 61.9 | 26.1 | 23.1 | 29.3 | 20.6 | 17.8 | 23.7 |
| Age |  |  |  |  |  |  |  |  |  |
| 16-34 | 56.8 | 52.4 | 61.1 | 26.1 | 22.4 | 30.2 | 21.3 | 17.9 | 25.2 |
| 35-49 | 56.0 | 49.6 | 62.2 | 25.0 | 20.0 | 30.9 | 23.7 | 18.6 | 29.8 |
| 50-59 | 66.8 | 59.4 | 73.5 | 29.2 | 22.7 | 36.6 | 21.4 | 15.6 | 28.6 |
| 60-69 | 67.1 | 57.5 | 75.5 | 32.0 | 23.5 | 42.0 | 26.7 | 18.8 | 36.3 |
| 70 + | 62.0 | 41.8 | 78.7 | 40.1 | 22.5 | 60.7 | 26.2 | 12.8 | 46.2 |
|  | | | | | | | | | |
|  | Chronic Cough | | | Breathlessness | | | Chronic Bronchitis | | |
|  | % | 95%CI | | % | 95%CI | | % | 95%CI | |
|  |  | Min | Max |  | Min | Max |  | Min | Max |
| Total | 20.5 | 18.2 | 22.9 | 19.5 | 17.1 | 22.2 | 5.1 | 3.8 | 6.6 |
| Sex |  |  |  |  |  |  |  |  |  |
| Men | 19.2 | 15.6 | 23.4 | 19.0 | 15.2 | 23.4 | 5.5* | 3.6 | 8.4 |
| Women | 21.8 | 19.0 | 24.8 | 20.1 | 17.6 | 22.9 | 4.6* | 3.3 | 6.3 |
| Age |  |  |  |  |  |  |  |  |  |
| 16-34 | 14.2* | 11.6 | 17.3 | 17.9 | 14.7 | 21.7 | 3.0* | 1.7 | 5.0 |
| 35-49 | 25.8* | 20.4 | 32.1 | 19.6 | 15.1 | 25.1 | 5.0* | 2.8 | 8.8 |
| 50-59 | 28.1* | 21.7 | 35.4 | 22.7 | 16.9 | 29.8 | 9.2* | 5.3 | 15.6 |
| 60-69 | 30.5* | 22.4 | 40.1 | 23.5 | 16.4 | 32.5 | 10.3* | 5.5 | 18.4 |
| 70 + | 20.8* | 8.8 | 41.9 | 20.2 | 8.7 | 40.3 | n.p. | - | - |

*p<0.05 according to a global chi-squared test.

n.p. Not presented because fewer than 5 participants.

^A^ “At least one symptom” among cough (usual, not necessarily chronic), sputum (usual, not necessarily chronic) or wheezing (in the last 12 months).

Table A4. Prevalence of diagnosed respiratory conditions according to medical files, population of Nunavik aged 16 and over, 20177

|  | % | 95%CI | |
| --- | --- | --- | --- |
|  |  | Min | Max |
| Tuberculosis of unknown status (latent or active, undetermined in the medical file) | 16.0 | 13.8 | 18.5 |
| Latent tuberculosis infection | 13.0 | 11.3 | 15.0 |
| Active tuberculosis disease (in the past, not currently) | 5.4 | 4.2 | 6.9 |
| Chronic Obstructive Lung Disease (COPD)^A^ | 6.5 | 5.4 | 7.9 |
| Hospitalization for respiratory infection in childhood | 6.4 | 4.9 | 8.2 |
| Asthma^B^ | 3.9 | 2.8 | 5.4 |
| Lung cancer | No participant |  |  |

^A^ There were less than 5 cases of COPD among 16-34 years old while the prevalence of COPD was 14.1% among 35+ years old.

^B^ Prevalence of asthma was significantly higher among 16-34 years old (5.2%) than 35+ years old (2.5%).

Table A5. Prevalence of tuberculosis (latent, active or unknown status) according to medical files, population of Nunavik aged 16 and over, 2017

|  | Active tuberculosis disease | | | Latent tuberculosis infection | | | Tuberculosis of unknown status (latent or active) | | |
| --- | --- | --- | --- | --- | --- | --- | --- | --- | --- |
|  | % | 95%CI | | % | 95%CI | | % | 95%CI | |
|  |  | Min | Max |  | Min | Max |  | Min | Max |
| Total | 5.4 | 4.2 | 6.9 | 13.0 | 11.3 | 15.0 | 16.0 | 13.8 | 18.5 |
| Age |  |  |  |  |  |  |  |  |  |
| 16-34 | 2.4* | 1.3 | 4.4 | 11.8* | 9.5 | 14.7 | 11.5* | 8.9 | 14.7 |
| 35-49 | 4.9* | 2.8 | 8.4 | 10.8* | 7.7 | 14.9 | 17.6* | 12.9 | 23.4 |
| 50-59 | 9.2* | 5.5 | 14.8 | 15.7* | 10.8 | 22.2 | 21.8* | 16.0 | 28.8 |
| 60-69 | 18.3* | 11.9 | 27.1 | 22.6* | 15.4 | 31.9 | 30.4* | 22.3 | 40.0 |
| 70 + | n.p. |  |  | n.p. |  |  | 20.2* | 7.9 | 42.7 |

*p<0.05 according to a global chi-squared test.

n.p. Not presented because fewer than 5 participants.

Table A6. Prevalence of prescribed medication according to medical files, population of Nunavik aged 16 and over, 2017

|  | % | 95%CI | |
| --- | --- | --- | --- |
|  |  | Min | Max |
| Prescription of short-action medication for asthma or COPD  (inhaled salbutamol or ipratropium bromide) | 1.8 | 1.3 | 2.6 |
| Prescription of medication for asthma  (inhaled corticosteroids, combined or not with a long-acting beta-agonist) | 1.1 | 0.6 | 1.8 |
| Prescription of medication for COPD  (inhaled long-acting anti-muscarinic and/or long-acting beta-agonist) | 1.2 | 0.7 | 2.0 |
| Prescription of medication for allergic rhinitis  (second-generation antihistamines) | 0.3 | 0.1 | 0.6 |

Table A7. Prevalence of allergic sensitization in the population of Nunavik aged 16 and over, 2017

|  | % | 95%CI | |
| --- | --- | --- | --- |
|  |  | Min | Max |
| Allergic Sensitization to at least two types among Dogs, Farinae, Pteronyssinus, Alternaria, Aspergillus | 4.4 | 3.3 | 5.8 |
| Allergic Sensitization to Dust Mites: Farinae and/or Pteronyssinus | 5.3 | 4.1 | 6.9 |
| Allergic Sensitization to Dust Mites: Farinae | 4.8 | 3.6 | 6.2 |
| Allergic Sensitization to Dust Mites: Pteronyssinus | 4.6 | 3.5 | 6.1 |
| Allergic Sensitization to Dogs | 2.8 | 1.9 | 4.1 |
| Allergic Sensitization to Moulds: Alternaria | 0.4 | 0.2 | 1.0 |
| Allergic Sensitization to Moulds: Aspergillus | 0.5 | 0.2 | 0.9 |

Supplementary Material B: Definition of variables

Table B.1 Definitions and categorization of study variables

| Variable | Data collection | Explanation (questions and categorisation) |
| --- | --- | --- |
| Tobacco smoking | Questionnaire | 1. We first established smoking status: daily, occasional, former or never smokers.   *“At the present time, do you smoke cigarettes daily, occasionally, or not at all?”*  For those who do not smoke at all at this time : “*Have you ever smoked cigarettes?”* ("*Never smoked; "Yes, but not a whole cigarette"; "Yes, at least one cigarette but fewer than 100 cigarettes (about 4 packs) in your lifetime"; "Yes, at least 100 cigarettes or more (about 4 packs) in your lifetime*").  Participants were considered “former smokers” if they had smoked at least 100 cigarettes.   1. Then we calculated pack-years for daily smokers:   Pack-year = (age – age_beginning) * (number_cigarettes /20)  Where age_beginning: At what age did you begin to smoke cigarettes daily?  Where number_cigarette: How many cigarettes do you smoke each day now?   1. Then we calculated pack-year for occasional smokers:   Pack-year = (age – age_beginning) * (number_cigarettes /20) * (number_days/30)  Where number_days : In the past month, about how many days have you smoked one or more cigarettes?   1. We finally combined daily and occasionally smokers according to their pack-year and created a variable with 4 mutually exclusive categories: current ≥ 15 pack-years; current < 15 pack-years; former, never. |
| Current ≥ 15 pack year |  |  |
| Current < 15 pack year |  |  |
| Former |  |  |
| Never |  |  |
| Electronic cigarette:  in past year | Questionnaire | *“In the past 12 months, have you used or tried an electronic cigarette (or e-cigarette/vapour), even just a few puffs? (Yes; No)”* |
| Recreational solvent: ever, at least once | Questionnaire | *“In your lifetime, have you tried to get high by sniffing glue, gasoline, propane, or any other solvent? (Yes; No)”* |
| Marijuana use:  daily/regular (> once/month) | Questionnaire | *“In the past 12 months, have you used or tried weed, pot, marijuana, grass, or hashish?”* The following categories were formed according to:   - Daily: "*Daily or almost daily"* [31.6%] - Regularly: "*2 or 3 times a month*" [2.8%], "*About once or twice a week*" [4.9%], "*3 to 4 times a week*" [8.8%] - Occasionally: "*About once a month*” [2.7%], "*3 to 11 times a year*" [3.6%] - Experimenter: “*Once or twice*"[9.0%] - Abstainer: "*Never”* [36.6%]   Daily and regular were combined, as were occasionally, experimenters and abstainers, assuming that a meaningful exposure should be necessary to cause respiratory problems. |
| Second-hand smoke exposure: almost daily | Questionnaire | “*Currently, how often are you exposed to second-hand smoke in your home? “*  Second-smoke exposure was not restricted to non-smokers because there were few non-smokers in the study. Instead, second-smoke exposure is adjusted for smoking in multivariate analysis.  Categories were dichotomized based on their frequency:   - Almost daily: "*Every day*" [14.2%], "*Nearly every day*" [13.0%] - Reference: "*Approximately once a week*" [5.7%], "*Approximately once a month*" [1.6%], "*Less than once a month"* [3.7%], "*Never*" [61.5%] |
| Body mass index | Physical measurements | Body-mass index was calculated as weight/(height)^2^ in kg/m^2^. For participants aged 16 and 17 years old, BMI was categorized as obesity, overweight, healthy or underweight according to reference curves for age and sex. For participants aged 18 and over, BMI was categorized in the same categories, but according to recognized fixed cut-offs. |
| ≥ 30 (obesity) |  |  |
| 25-30 (overweight) |  |  |
| 18.5-24.9 (healthy) |  |  |
| < 18,5 (underweight) |  |  |
| Marine animals & fish | Food-frequency questionnaire | Each food was assessed by the question, “*In the last 3 months, how often on average do you eat this food*? …”. Frequencies ("*never or less than once a month,” "1-3 times a month,” "once a week,” "2-6 times a week,” "once a day,” "2-3 times a day,” "4 times and more a da*y") were reported in weekly frequencies.  Marine animals and fish were: “*Beluga - Dried meat (nikku)”, “Beluga – Meat,” “Beluga - Misirak/Ursuk (blubber only)”, “Beluga - Mattaaq (skin and blubber)”, “Seal - Meat (fresh, cooked, frozen)”, “Seal - Meat (fresh, cooked, frozen)”, “Seal - Misirak/Ursuk (blubber only)”, “Seal – Liver,” “walrus meat, igunak,” “Dried fish (nikku, pitsik)”, “Lake trout (fresh, cooked or frozen, NOT dried)”, “Brook or sea trout, or salmon (fresh, cooked, canned or frozen, NOT dried)”, “Arctic char (fresh, cooked or frozen, NOT dried)”, “Pike or walleye”, “Other fish, e.g. Lake whitefish (Coregone), Sculpin (Ugly fish)”.*  Weekly frequencies for each food were summarized and categorized in rounded and approximately equal categories, according to the distribution in the population. Many categories have been used in the literature, but the cut-off of three times/week has been used in previous research and public health guidelines (1). |
| ≥ 7 times/week |  |  |
| 3-6 times/week |  |  |
| <3 times/week |  |  |
| Fruits/Vegetables: ≥5 times/day | Food frequency questionnaire | Each food was assessed by the question, “*In the last 3 months, how often on average do you eat this food?* …”. Frequencies ("*never or less than once a month,” "1-3 times a month,” "once a week,” "2-6 times a week,” "once a day,” "2-3 times a day,” "4 times and more a day*") were reported in daily frequencies.  Fruits and vegetables were: “*Wild berries,” “Fruits (orange, banana, apple, pear, store-bought berries, etc.). (Fresh or frozen)”, “Canned fruit”, “Applesauce, Fruit puree”, “Green, leafy vegetables (iceberg, romaine or leaf lettuce, spinach)”, “Carrots”, “Broccoli, cauliflower, cabbage”, “Tomatoes (whole or canned) or V8 juice”, “Other vegetables (pepper (green, red, yellow), onions, corn, cucumber, celery, mushrooms, mixed vegetables)(fresh, frozen, canned)”.*  The cut-off of 5 times/day is largely used public health’s objective (Agudo, 2004). |
| Vitamin D: sufficient blood level | Blood test | Level of 25-OH vitamin D were measured in serum with Roche Technic. Sufficient value is the lower limit of normal values, i.e., 50 nmol/L. |
| Respiratory infection during childhood | Review of medical files | Reviewers searched files for respiratory infection before the age of 5, which requires hospitalization. Hospitalization included only stays in the two Nunavik’s hospitals located in Kuujjuak and Puvirnituq and transfers to Québec’s southern hospitals. Children who stayed in local health centres are not counted as hospitalized, even if they spent more than 24 hours in the health centre. |
| Active TB in the past | Review of medical files | Reviewers searched for different mention of tuberculosis and noted status (active disease versus latent infection) as written in the files. Active TB included active tuberculosis/TB, pulmonary tuberculosis/TB, TB meningitis, primo infection. Mention of tuberculosis without indication of the status was considered as missing data. Latent infection was considered as no active TB since it could not cause respiratory problems. |
| Allergic sensitization to dogs | Blood test | Blood samples were analyzed with fluoro-enzyme immunoassays (ImmunoCAP^TM^) to detect the presence of dog specific IgE. The threshold for sensitization was the level of detection of the test (0.34 kUA/L). This test was performed only if participant had elevated blood total IgE (> 100 kU/L), representing 24.5% of the population. Other participants, who had normal blood total IgE and were not tested for specific IgE, were assumed not to be sensitized. |
| Allergic sensitization to dust mites | Blood test | Blood samples were analyzed with fluoro-enzyme immunoassays (ImmunoCAP^TM^) to detect the presence Dermatophagoides pteronyssinus and Dermatophagoides farina. The threshold for sensitization was the level of detection of the test (0.34 kUA/L). This test was performed only if participant had elevated blood total IgE (> 100 kU/L), representing 24.5% of the population. Other participants, who had normal blood total IgE and were not tested for specific IgE, were assumed not to be sensitized. |
| High blood total IgE  (≥ 100 kU/L) | Blood test | Level of total immunoglobulin E (IgE) was measured in plasma. |
| Housing crowding (> 1 person-per-room) | Questionnaire and administrative data | Housing crowding is defined by “person-per-room” as used in Canadian Census (Statistic Canada, 2019 June 24).  Person-per-room = number_people/(number_bedroom+2)  Where number_people: “*Including you, how many people usually live in your home? Think about all persons who usually live in your home, even if they are temporarily away. Do not count people who have a usual home elsewhere.”*  Where number_bedroom was obtained from Kativik Municipal Housing Bureau. Two additional rooms were added for kitchen and living room. |
| Housing in need of major repairs | Questionnaire | “*Is your current house in need of repair?”* Categories were dichotomized based on their frequency:   - Need of major repairs: "*Yes, major repairs are needed (defective plumbing or electrical wiring, structural repairs to walls, floors or ceiling, etc.)."* - No need of major repairs: "*No, only regular maintenance is needed (painting, furnace cleaning, etc*.)" or *"Yes, minor repairs are needed (missing or loose floor tiles, bricks or shingles, defective steps, railing or siding, etc.)."* |
| Food security | Questionnaire | The three categories of food security are obtained from Household Food Security Survey Module, adults scale used by Health Canada (Health Canada, 2019 June 24).  Questions adapted to Inuit reality: “Money” replaced by “resources.” Questions were :   - *In the last year, how often did you worry that the food in your house would run out before you had the resources to get more (e.g., money to buy food, equipment to hunt, fish or gather food, social connections to get food from, etc.)?* - *How often did it happen that the food in your house just didn't last and you didn't have resources to get more?* - *How often were you not able to eat healthy foods because you didn't have resources to get them?* - *Did you ever cut the size of your meals or skip meals because you didn't have resources to get food? How often did this happen?* - *Did you ever eat less than you felt you should because you didn't have resources to get food?* - *Were you ever hungry but didn't eat because you didn't have resources to get food?* - *Did you lose weight because you didn't have resources to get food?* - *Did you ever not eat for a whole day because you didn't have resources to get food?* - *How often did this happen?*   Food security status was food secure if 0-1 affirmative response; moderately food insecure if 2-5 affirmative responses; and severely food insecure if ≥6 affirmative responses. |
| Food secure |  |  |
| Moderately food  insecure |  |  |
| Severely food insecure |  |  |
| Personal income | Questionnaire | “*What is your best estimate of your total personal income, before taxes and other deductions, from all sources in the past 12 months?”*  Canadian Institute for Health Information recommends using income quintiles (Canadian Institute for Health Information, 2018) but it was not possible with this questionnaire. Categories were combined in 5 categories that correspond approximately to the quintiles of total personal income before taxes in Québec in 2017 (Institut de la statistique du Québec, 2019 June 24). In addition, the distribution of income in much lower in Nunavik than in the rest of Québec, so using quintiles in Nunavik would still have resulted in relatively “low” income among higher categories.   - *≥ 60,000$: "$60,000 to less than $80,000,” "$80,000 or more"* - *40,000 - 59,999$: "$40,000 to less than $60,000"* - *25,000 - 39,999$:"$25,000 to less than $40,000"* - *15,000 - 24,999$:"$20,000 to less than $25,000", "$15,000 to less than $20,000"* - *< 15,000$: "Less than $15,000"* |
| ≥ 60,000$ |  |  |
| 40,000 - 59,999$ |  |  |
| 25,000 - 39,999$ |  |  |
| 15,000 - 24,999$ |  |  |
| < 15,000$ |  |  |
| School level | Questionnaire | “*What is the highest grade you have completed? “*  Categories were combined as recommended by CIHI but post-secondary categories were combined because of small numbers (Canadian Institute for Health Information, 2018).   - Post-secondary: "Graduated From CEGEP/college,” "Some University, but not graduated,” "Graduated from University." - High school completed: "Grade 11/secondary 5 (graduated)", "Some CEGEP/ college, but not graduated." - Some high school or less: "Grade 1,” "Grade 2,” "Grade 3,” "Grade 4,” "Grade 5,” "Grade 6,” "Grade 7/secondary 1,” "Grade 8/secondary 2,” "grade 9/secondary 3,” "Grade 10/secondary 4." |
| Post-secondary |  |  |
| High school completed |  |  |
| Some high school or  less |  |  |
| Going on the land: often | Questionnaire | “*From the Spring until now, how often did you go on the land?”*  Categories were dichotomized based on their frequency:   - "*Often"[*43.6%] - "*Occasionally"* [42.9%] or "*Never"* [13.4%] |
| Traditional activities: in the last year | Questionnaire | *“Over the past 12 months, which of the following activities did you participate in? Harvesting or traditional activities (e.g., hunting, going on the land, sewing) (Yes; No)”* |

[%] When answers were combined, weighted proportion of each answer in the total population before the imputation is provided in squared brackets.

**References from Table B1**

Agudo, A. & Joint FAO/WHO Workshop on Fruit and Vegetables for Health. (2005). Measuring intake of fruit and vegetables - Background Paper for the Joint FAO/WHO Workshop on Fruit and Vegetables for Health. World Health Organization. <https://apps.who.int/iris/handle/10665/43144>

Canadian Institute for Health Information (CIHI). (2018). In Pursuit of Health Equity - Defining Stratifiers for Measuring Health Inequality - A Focus on Age, Sex, Gender, Income, Education and Geographic Location. <https://www.cihi.ca/en/measuring-health-inequalities-a-toolkit>

Ferkol, T. et Schraufnagel, D. (2014). The global burden of respiratory disease. Annals of the American Thoracic Society, 11(3), 404‑406. <https://doi.org/10.1513/AnnalsATS.201311-405PS>

Health Canada. (2019, June 24). Determining Food Security Status. <https://www.canada.ca/en/health-canada/services/food-nutrition/food-nutrition-surveillance/health-nutrition-surveys/canadian-community-health-survey-cchs/household-food-insecurity-canada-overview/determining-food-security-status-food-nutrition-surveillance-health-canada.html#as>

Institut de la statistique du Québec (ISQ). (2019, June 24). Indicateurs par quintile, revenu total, particuliers (16 ans et plus), Québec, 2017. <http://www.stat.gouv.qc.ca/statistiques/conditions-vie-societe/revenu/inegalite-revenu/mod3_p_1_2_2_0_.htm>

Statistic Canada. (2019, June 24). Housing suitability of private household. <http://www23.statcan.gc.ca/imdb/p3Var.pl?Function=DEC&Id=100731>

Supplementary Material C: Additional Results

Table C1. Description of study variables, before and after multiple imputation

|  | Before multiple imputation | | | After multiple imputation (pooled data) | |
| --- | --- | --- | --- | --- | --- |
|  | % Missing Data | N | % (95% CI) | N | % (95% CI) |
| Tobacco smoking |  |  |  |  |  |
| Never | 5.1 | 135 | 10.3 (8.4-12.1) | 143 | 10.4 (8.5-12.3) |
| Former |  | 143 | 10.8 (8.9-12.7) | 149 | 10.7 (8.8-12.5) |
| Current, < 15 pack year |  | 674 | 53.7 (50.5-56.9) | 713 | 54.0 (50.8-57.2) |
| Current, ≥ 15 pack year |  | 278 | 25.2 (22.3-28.1) | 291 | 24.9 (22.1-27.8) |
| Electronic cigarette: in past year | 2.1 | 142 | 12.2 (10.0-14.3) | 145 | 12.2 (10.0-14.4) |
| Recreational solvent: ever, at least once | 2.0 | 320 | 29.1 (26.2-32.1) | 327 | 29.1 (26.1-32.1) |
| Marijuana use: daily/regular (> once/month) | 2.2 | 574 | 48.2 (45.0-51.3) | 588 | 48.3 (45.1-51.5) |
| Second-hand smoke exposure: almost daily | 1.9 | 408 | 33.1 (30.1-36.1) | 417 | 33.2 (30.1-36.2) |
| Body mass index | 8.4 |  |  |  |  |
| < 18,5 (underweight) |  | 27 | 1.6 (0.9-2.3) | 31 | 1.8 (1.0-2.5) |
| 18.5-24.9 (healthy) |  | 472 | 42.7 (39.4-46) | 514 | 42.5 (39.3-45.7) |
| 25-30 (overweight) |  | 317 | 25.8 (23.0-28.6) | 346 | 25.9 (23.1-28.6) |
| ≥ 30 (obesity) |  | 371 | 29.9 (26.9-32.8) | 405 | 29.9 (27.0-32.8) |
| Marine mammals & fish |  |  |  |  |  |
| <3 times/week | 11.4 | 348 | 28.9 (25.9-31.9) | 392 | 28.9 (25.9-31.8) |
| 3-6 times/week |  | 349 | 29.8 (26.8-32.9) | 393 | 29.9 (26.9-32.9) |
| ≥ 7 times/week |  | 451 | 41.3 (37.9-44.7) | 511 | 41.2 (37.9-44.4) |
| Fruits/Vegetables: ≥5 times/day | 11.4 | 178 | 15.1 (12.7-17.5) | 200 | 14.9 (12.6-17.3) |
| Vitamin D: sufficient blood level | 6.0 | 857 | 68.4 (65.2-71.5) | 387 | 67.9 (64.8-71) |
| Respiratory infection during childhood | 6.6 | 77 | 6.5 (4.9-8.1) | 86 | 6.8 (5.1-8.5) |
| Active TB in the past | 21.2 | 70 | 6.4 (4.7-8.0) | 103 | 7.3 (5.4-9.1) |
| Allergic sensitization to dogs | 6.0 | 38 | 2.8 (1.7-3.8) | 43 | 3.1 (1.9-4.2) |
| Allergic sensitization to dust mites | 6.0 | 72 | 5.4 (4.0-6.9) | 80 | 5.8 (4.3-7.4) |
| High blood total IgE (≥ 100 kU/L) | 6.3 | 320 | 24.5 (21.8-27.3) | 347 | 25.5 (22.7-28.4) |
| Housing crowding (> 1 person-per-room) | 9.2 | 386 | 32.3 (29.2-35.3) | 423 | 32.0 (29.0-35.1) |
| Housing in need of major repairs | 5.9 | 236 | 19.1 (16.6-21.7) | 251 | 19.1 (16.6-21.7) |
| Food security |  |  |  |  |  |
| Food secure | 7.1 | 416 | 33.5 (30.4-36.6) | 441 | 32.9 (29.9-35.9) |
| Moderately food insecure |  | 566 | 48.1 (44.9-51.4) | 610 | 48.1 (44.8-51.3) |
| Severely food insecure |  | 222 | 18.4 (15.9-20.9) | 245 | 19.0 (16.5-21.6) |
| Personal income |  |  |  |  |  |
| < 15,000$ | 13.9 | 450 | 40.9 (37.5-44.2) | 533 | 41.2 (38.0-44.4) |
| 15,000 - 24,999$ |  | 216 | 19.3 (16.7-22.0) | 251 | 19.2 (16.6-21.9) |
| 25,000 - 39,999$ |  | 138 | 13.0 (10.6-15.3) | 161 | 13.0 (10.8-15.3) |
| 40,000 - 59,999$ |  | 136 | 13.1 (10.7-15.5) | 154 | 13.0 (10.6-15.3) |
| ≥ 60,000$ |  | 176 | 13.7 (11.5-15.9) | 198 | 13.6 (11.4-15.7) |
| School level |  |  |  |  |  |
| Post-secondary | 3.0 | 84 | 5.3 (4.0-6.5) | 91 | 5.6 (4.3-6.8) |
| High school completed |  | 287 | 23.8 (21.1-26.6) | 292 | 23.6 (20.9-26.3) |
| Some high school or less |  | 886 | 70.9 (68.0-73.8) | 913 | 70.9 (68.0-73.7) |
| Traditional activities: in the last year | 0.4 | 1163 | 89.5 (87.5-91.4) | 1167 | 89.4 (87.5-91.4) |
| Going on the land: often | 0.4 | 548 | 43.6 (40.5-46.7) | 550 | 43.6 (40.4-46.7) |

Numbers (N) are rounded and unweighted to represent the sample while proportions (%) are weighted to represent Nunavik’s population.

Table C2. Age/sex-adjusted logistic models for wheezing, population of Nunavik aged 16 and over, 2017

|  | Total population (N = 1,258) | 16-34 years old (N = 608) | 35 years and older (N = 650) | Men (N = 439) | Women (N = 819) |
| --- | --- | --- | --- | --- | --- |
|  | OR (95% CI) | OR (95% CI) | OR (95% CI) | OR (95% CI) | OR (95% CI) |
| Sex: men (vs. women) | 1.09 (0.82-1.45) | 1.14 (0.75-1.73) | 1.05 (0.71-1.54) |  |  |
| Tobacco smoking |  |  |  |  |  |
| Never | ref | 0.04 (0.00-0.33)* | ref | ref | ref |
| Former | 2.56 (1.30-5.03)* | 1.67 (0.76-3.66) | 1.20 (0.58-2.51) | 3.22 (1.22-8.53)* | 1.99 (0.76-5.23) |
| Current, < 15 pack year | 1.84 (1.06-3.19)* | ref^A^ | 0.95 (0.49-1.86) | 1.68 (0.8-3.510) | 2.03 (0.86-4.76) |
| Current, ≥15 pack year | 3.26 (1.74-6.10)* | 3.68 (1.41-9.64)* | 1.45 (0.77-2.74) | 2.97 (1.25-7.07)* | 3.82 (1.50-9.75)* |
| Recreational solvent: ever, at least once | 1.52 (1.09-2.12)* | 3.01 (1.85-4.91)* | 0.86 (0.55-1.33) | 1.32 (0.79-2.21) | 1.84 (1.24-2.71)* |
| Marijuana use: daily/regular (> once/month) | 1.22 (0.90-1.64) | 1.62 (1.04-2.54)* | 0.91 (0.61-1.37) | 1.05 (0.66-1.67) | 1.44 (1.01-2.04)* |
| Second-hand smoke exposure: almost daily | 1.34 (0.99-1.82) | 1.41 (0.89-2.23) | 1.28 (0.85-1.93) | 1.38 (0.86-2.22) | 1.31 (0.91-1.88) |
| Body mass index (ref : 18.5-24.9) |  |  |  |  |  |
| < 18,5 (underweight) | 1.76 (0.70-4.44) | 1.38 (0.34-5.56) | 2.27 (0.61-8.40) | 0.95 (0.09-9.63) | 2.09 (0.80-5.47) |
| 25-30 (overweight) | 1.28 (0.87-1.88) | 1.52 (0.87-2.65) | 1.06 (0.63-1.80) | 1.43 (0.79-2.59) | 1.12 (0.71-1.76) |
| ≥ 30 (obesity) | 1.50 (1.04-2.15)* | 1.18 (0.67-2.07) | 1.74 (1.08-2.82)* | 1.64 (0.92-2.91) | 1.34 (0.87-2.06) |
| Marine mammals & fish (ref : < 3 times/week) | |  |  |  |  |
| 3-7 times/week | 1.34 (0.91-1.98) | 1.70 (0.98-2.96) | 1.04 (0.61-1.79) | 1.33 (0.69-2.56) | 1.37 (0.87-2.15) |
| ≥ 7 times/week | 1.75 (1.22-2.51)* | 2.01 (1.17-3.46)* | 1.56 (0.96-2.54) | 2.09 (1.16-3.77)* | 1.46 (0.94-2.25) |
| Fruits/Vegetables: ≥ 5 times/day | 1.55 (1.05-2.29)* | 1.11 (0.64-1.92) | 2.10 (1.21-3.64)* | 1.43 (0.72-2.86) | 1.67 (1.07-2.61)* |
| Vitamin D: sufficient blood level | 0.84 (0.59-1.21) | 1.10 (0.70-1.72) | 0.55 (0.32-0.94)* | 0.97 (0.55-1.71) | 0.70 (0.47-1.06) |
| Respiratory infection during childhood | 1.63 (0.95-2.82) | 0.97 (0.31-3.08) | 1.94 (1.04-3.62)* | 1.23 (0.48-3.16) | 2.02 (1.08-3.78)* |
| Allergic sensitization to dogs | 2.50 (1.14-5.53)* | 2.37 (0.77-7.33) | 2.71 (0.92-7.96) | 4.64 (1.08-19.93)* | 1.40 (0.56-3.50) |
| High blood total IgE | 1.15 (0.83-1.59) | 0.91 (0.53-1.57) | 1.36 (0.90-2.07) | 1.05 (0.62-1.75) | 1.29 (0.88-1.90) |
| Housing crowding (> 1 person-per-room) | 0.54 (0.38-0.77)* | 0.63 (0.39-1.00) | 0.44 (0.27-0.74)* | 0.60 (0.35-1.05)* | 0.48 (0.32-0.72)* |
| Housing in need of major repairs | 1.29 (0.89-1.85) | 1.20 (0.69-2.10) | 1.36 (0.85-2.19) | 0.93 (0.51-1.69) | 1.84 (1.22-2.79)* |
| Food security (ref: severely food insecure) |  |  |  |  |  |
| Food secure | 0.55 (0.37-0.84)* | 0.65 (0.34-1.23) | 0.47 (0.27-0.82)* | 0.65 (0.34-1.25) | 0.46 (0.28-0.75)* |
| Moderately food insecure | 0.64 (0.43-0.94)* | 0.85 (0.48-1.49) | 0.48 (0.28-0.82)* | 0.70 (0.39-1.27) | 0.57 (0.36-0.90)* |
| Personal income (ref: < 15,000$) |  |  |  |  |  |
| 15,000 - 24,999$ | 1.11 (0.72-1.72) | 1.16 (0.64-2.13) | 1.03 (0.56-1.89) | 1.34 (0.68-2.62) | 0.92 (0.54-1.56) |
| 25,000 - 39,999$ | 1.07 (0.66-1.73) | 1.65 (0.85-3.21) | 0.64 (0.33-1.26) | 0.93 (0.44-1.93) | 1.32 (0.73-2.39) |
| 40,000 - 59,999$ | 0.88 (0.50-1.54) | 0.79 (0.30-2.11) | 0.87 (0.44-1.74) | 0.97 (0.41-2.27) | 0.81 (0.40-1.63) |
| ≥60,000 $ | 1.59 (1.00-2.55) | 2.75 (1.06-7.15)* | 1.26 (0.73-2.17) | 2.04 (0.93-4.44) | 1.31 (0.75-2.30) |
| School attainment (ref: high school not completed) | |  |  |  |  |
| Post-secondary | 1.16 (0.66-2.07) | 0.53 (0.12-2.32) | 1.31 (0.70-2.46) | 1.84 (0.58-5.79) | 0.91 (0.47-1.75) |
| High school completed | 0.95 (0.67-1.34) | 0.96 (0.59-1.54) | 0.93 (0.56-1.54) | 0.69 (0.39-1.20) | 1.33 (0.88-2.00) |
| Traditional activities: in the last year | 1.33 (0.81-2.18) | 1.74 (0.85-3.59) | 1.05 (0.53-2.06) | 1.13 (0.57-2.22) | 1.78 (0.88-3.58) |
| Going on the land: often | 1.16 (0.87-1.55) | 1.36 (0.88-2.08) | 1.00 (0.67-1.48) | 1.16 (0.73-1.85) | 1.18 (0.84-1.65) |

In each population, OR are adjusted for age (16-24, 25-34, 35-49, 50-59, >= 60 years old) and sex.

Other variables not presented because p-value always > 0.2 (with small OR) are electronic cigarette, allergic sensitization to dust mites, and history of active TB in the past.

^A^ The reference category is different for young adults because the number of cases among never smokers was too small to yield reliable estimates.

*p<0.05

Table C3. Age/sex-adjusted logistic models for chronic cough, population of Nunavik aged 16 and over, 2017

|  | Total population (N= 1,257) | 16-34 years old (N = 609) | 35 years and older (N = 648) | Men (N = 437) | Women (N = 820) |
| --- | --- | --- | --- | --- | --- |
|  | OR (95% CI) | OR (95% CI) | OR (95% CI) | OR (95% CI) | OR (95% CI) |
| Sex: men versus women | 0.87 (0.63-1.20) | 0.58 (0.33-1) | 1.11 (0.74-1.67) |  |  |
| Tobacco smoking |  |  |  |  |  |
| Never | ref | 0.16 (0.04-0.65)* | ref | ref | ref |
| Former | 1.03 (0.46-2.28) |  | 0.77 (0.33-1.80) | 0.85 (0.26-2.81) | 1.06 (0.37-3.02) |
| Current, < 15 pack year | 2.43 (1.30-4.53)* | ref | 1.50 (0.72-3.14) | 2.74 (1.10-6.84)* | 2.11 (0.91-4.91) |
| Current, ≥15 pack year | 4.05 (2.09-7.83)* | 1.51 (0.49-4.66) | 2.84 (1.43-5.61)* | 3.61 (1.42-9.20)* | 4.74 (1.96-11.46)* |
| Electronic cigarette | 1.43 (0.89-2.28) | 1.45 (0.80-2.63) | 1.49 (0.69-3.21) | 1.10 (0.52-2.31) | 2.00 (1.15-3.46)* |
| Recreational solvent: ever, at least once | 1.32 (0.92-1.89)* | 2.05 (1.18-3.57)* | 1.01 (0.64-1.58) | 1.28 (0.73-2.23) | 1.24 (0.81-1.91) |
| Marijuana use: daily/regular (> once/month) | 1.70 (1.22-2.37)* | 1.55 (0.92-2.60)* | 1.76 (1.14-2.70)* | 1.43 (0.83-2.46) | 1.87 (1.27-2.74)* |
| Second-hand smoke exposure: almost daily | 1.65 (1.18-2.30)* | 1.29 (0.75-2.22) | 1.90 (1.24-2.92)* | 1.76 (1.02-3.02)* | 1.64 (1.11-2.42)* |
| Body mass index (vs. 18.5-24.9) |  |  |  |  |  |
| < 18,5 (underweight) | 2.28 (0.85-6.13) | 2.66 (0.67-10.53) | 1.90 (0.49-7.40) | 1.11 (0.06-21.35) | 2.71 (1.00-7.35)* |
| 25-30 (overweight) | 0.79 (0.52-1.21) | 0.96 (0.49-1.89) | 0.69 (0.40-1.17) | 0.83 (0.42-1.64) | 0.76 (0.46-1.25) |
| ≥ 30 (obesity) | 0.72 (0.48-1.09) | 0.72 (0.36-1.42) | 0.69 (0.42-1.14) | 0.61 (0.30-1.22) | 0.82 (0.51-1.31) |
| Marine mammals & fish (vs < 3 times/week) |  |  |  |  |  |
| 3-7 times/week | 1.25 (0.80-1.95) | 1.36 (0.69-2.69) | 1.28 (0.71-2.30) | 1.33 (0.58-3.03) | 1.24 (0.76-2.04) |
| ≥ 7 times/week | 1.94 (1.28-2.93)* | 3.06 (1.64-5.71)* | 1.52 (0.89-2.59) | 2.46 (1.19-5.11)* | 1.62 (1.01-2.60)* |
| Fruits/Vegetables: ≥ 5 times/day | 1.39 (0.90-2.17) | 1.42 (0.76-2.67) | 1.35 (0.75-2.44) | 1.78 (0.83-3.81) | 1.17 (0.71-1.91) |
| Respiratory infection during childhood | 2.52 (1.44-4.40)* | 2.44 (0.79-7.56) | 2.67 (1.41-5.06)* | 2.71 (1.05-6.98)* | 2.58 (1.35-4.95)* |
| Active TB in the past | 2.11 (1.17-3.80)* | 0.51 (0.07-4.03) | 2.65 (1.35-5.20)* | 1.89 (0.75-4.76) | 2.45 (1.20-5.01)* |
| Allergic sensitization to dogs | 0.52 (0.17-1.60) | 1.13 (0.32-3.90) | 0.16 (0.02-1.51) | No case | 0.96 (0.32-2.84) |
| Housing crowding (> 1 person-per-room) | 1.58 (1.10-2.25)* | 2.15 (1.28-3.62)* | 1.24 (0.76-2.04) | 1.67 (0.91-3.07) | 1.54 (1.03-2.29)* |
| Housing in need of major repairs | 1.81 (1.23-2.66)* | 2.24 (1.23-4.08)* | 1.58 (0.96-2.59) | 1.98 (1.05-3.73)* | 1.70 (1.09-2.65)* |
| Food security (vs severely food insecure) |  |  |  |  |  |
| Food secure | 0.26 (0.16-0.41)* | 0.24 (0.11-0.52)* | 0.26 (0.14-0.46)* | 0.22 (0.10-0.49)* | 0.27 (0.16-0.46)* |
| Moderately food insecure | 0.54 (0.36-0.82)* | 0.64 (0.34-1.20) | 0.48 (0.28-0.83)* | 0.61 (0.32-1.18) | 0.49 (0.30-0.78)* |
| Personal income (vs < 15,000$) |  |  |  |  |  |
| 15,000 - 24,999$ | 1.75 (1.14-2.69)* | 1.71 (0.93-3.14) | 1.77 (0.96-3.26) | 1.88 (0.91-3.89) | 1.70 (1.00-2.90) |
| 25,000 - 39,999$ | 1.08 (0.61-1.91) | 1.05 (0.41-2.69) | 1.10 (0.52-2.29) | 1.43 (0.60-3.42) | 0.79 (0.39-1.61) |
| 40,000 - 59,999$ | 1.04 (0.56-1.92) | 0.76 (0.17-3.34) | 1.10 (0.54-2.27) | 1.17 (0.45-3.07) | 0.91 (0.45-1.84) |
| ≥ 60,000 $ | 0.86 (0.49-1.50) | 3.04 (1.01-9.14)* | 0.65 (0.34-1.21) | 0.79 (0.28-2.18) | 0.91 (0.48-1.72) |
| School attainment (vs high school not completed) | |  |  |  |  |
| Post-secondary | 0.82 (0.45-1.52) | 0.70 (0.14-3.48) | 0.86 (0.45-1.66) | 0.70 (0.20-2.48) | 0.95 (0.48-1.89) |
| High school completed | 0.67 (0.44-1.01) | 0.81 (0.45-1.47) | 0.57 (0.32-1.01) | 0.59 (0.29-1.18) | 0.77 (0.48-1.25) |
| Traditional activities: in the last year | 0.62 (0.39-0.99)* | 1.13 (0.50-2.52) | 0.45 (0.24-0.84) | 0.58 (0.30-1.14) | 0.74 (0.40-1.39) |
| Going on the land: often | 0.64 (0.46-0.90)* | 0.73 (0.43-1.23) | 0.60 (0.39-0.92) | 0.62 (0.35-1.10) | 0.69 (0.48-1.00) |

OR are adjusted for age categories (16-24, 25-34, 35-49, 50-59, ≥ 60 when relevant) and sex.

Other variables not presented because p-value always > 0.2 (with small OR) are allergic sensitization to dust mites, sufficient blood level of vitamin D, and high blood total IgE.

^A^ The reference category is different for young adults because the number of cases among never and former smokers was too small to yield reliable estimates. The two categories were combined because no never smoker had chronic cough.

*p<0.05

Table C4. Age/sex-adjusted logistic models for airway obstruction^A^, population of Nunavik aged 16 and over, 2017

|  | Total population (N = 1,088) | 16-34 years old (N = 535) | 35 years and older (N = 553) | Men (N = 376) | Women (N = 712) |
| --- | --- | --- | --- | --- | --- |
|  | OR (95% CI) | OR (95% CI) | OR (95% CI) | OR (95% CI) | OR (95% CI) |
| Sex: men versus women | 1.54 (1.04-2.27)* | 1.33 (0.78-2.28) | 1.80 (1.01-3.19)* |  |  |
| Tobacco smoking |  |  |  |  |  |
| Never | ref | 0.66 (0.24-1.78) | ref | ref | ref |
| Former | 1.49 (0.57-3.91) | 0.73 (0.20-2.62) | 1.79 (0.52-6.09) | 1.85 (0.59-5.82) | 1.59 (0.26-9.56) |
| Current, < 15 pack year | 1.54 (0.73-3.23) | ref^B^ | 1.32 (0.41-4.22) | 1.04 (0.42-2.56) | 4.86 (1.2-19.68)* |
| Current, ≥15 pack year | 4.01 (1.80-8.91)* | 1.91 (0.60-6.09) | 4.37 (1.61-11.88)* | 2.67 (1.01-7.08)* | 14.10 (3.34-59.45)* |
| Recreational solvent: ever, at least once | 1.17 (0.73-1.87) | 0.79 (0.41-1.54) | 1.04 (0.55-1.96) | 1.13 (0.58-2.20) | 1.14 (0.64-2.03) |
| Marijuana use: daily/regular (> once/month) | 2.00 (1.30-3.07)* | 2.35 (1.34-4.12)* | 1.68 (0.88-3.22) | 1.64 (0.86-3.11) | 2.56 (1.57-4.17)* |
| Second-hand smoke exposure: almost daily | 1.39 (0.91-2.11) | 0.96 (0.52-1.78) | 2.01 (1.10-3.68)* | 1.44 (0.78-2.64) | 1.27 (0.75-2.15) |
| Body mass index (vs 18.5-24.9) |  |  |  |  |  |
| < 18,5 (underweight) | 4.35 (1.33-14.28)* | 9.78 (1.88-50.75)* | 2.16 (0.42-11.01) | 1.51 (0.1-22.26) | 5.85 (1.74-19.64)* |
| 25-30 (overweight) | 0.96 (0.60-1.55) | 1.15 (0.59-2.26) | 0.73 (0.37-1.43) | 1.08 (0.54-2.15) | 0.78 (0.43-1.43) |
| ≥ 30 (obesity) | 0.48 (0.26-0.9)* | 0.43 (0.19-0.98)* | 0.46 (0.20-1.08) | 0.55 (0.21-1.43) | 0.38 (0.19-0.76)* |
| Marine mammals & fish (vs < 3 times/week) |  |  |  |  |  |
| 3-7 times/week | 1.18 (0.70-1.99) | 1.12 (0.54-2.33) | 1.21 (0.59-2.45) | 0.75 (0.34-1.63) | 2.19 (1.17-4.12)* |
| ≥7 times/week | 0.90 (0.53-1.53) | 0.75 (0.35-1.60) | 1.11 (0.54-2.28) | 0.81 (0.39-1.71) | 1.11 (0.57-2.14) |
| Fruits/Vegetables: ≥ 5 times/day | 1.00 (0.51-1.96) | 0.83 (0.39-1.76) | 1.16 (0.41-3.28) | 1.16 (0.42-3.20) | 0.87 (0.42-1.80) |
| Vitamin D: sufficient blood level | 0.81 (0.50-1.32) | 1.08 (0.60-1.92) | 0.54 (0.25-1.16) | 0.82 (0.41-1.64) | 0.85 (0.48-1.50) |
| Respiratory infection during childhood | 1.90 (0.82-4.39) | 1.94 (0.43-8.63) | 1.90 (0.71-5.13) | 2.07 (0.58-7.32) | 1.64 (0.57-4.70) |
| Active TB in the past | 1.04 (0.43-2.52) | 0.17 (0.02-1.67) | 1.41 (0.53-3.77) | 0.75 (0.20-2.89) | 1.37 (0.46-4.12) |
| Allergic sensitization to dogs | 1.86 (0.77-4.44) | 1.30 (0.35-4.78) | 2.52 (0.83-7.63) | 1.43 (0.38-5.40) | 2.37 (0.84-6.71) |
| Housing crowding (> 1 person-per-room) | 0.91 (0.57-1.44) | 1.20 (0.66-2.19) | 0.59 (0.29-1.20) | 0.71 (0.35-1.47) | 1.23 (0.73-2.07) |
| Housing in need of major repairs | 0.90 (0.55-1.48) | 1.14 (0.57-2.27) | 0.69 (0.34-1.39) | 0.80 (0.39-1.63) | 1.09 (0.58-2.04) |
| Food security (vs severely food insecure) |  |  |  |  |  |
| Food secure | 0.76 (0.43-1.34) | 0.72 (0.32-1.65) | 0.77 (0.36-1.66) | 0.76 (0.34-1.69) | 0.84 (0.39-1.78) |
| Moderately food insecure | 0.75 (0.45-1.26) | 1.02 (0.46-2.22) | 0.53 (0.26-1.05) | 0.56 (0.27-1.15) | 1.21 (0.59-2.45) |
| Personal income (vs < 15,000$) |  |  |  |  |  |
| 15,000 - 24,999$ | 1.22 (0.70-2.11) | 1.65 (0.80-3.39) | 0.77 (0.33-1.77) | 1.15 (0.48-2.74) | 1.37 (0.71-2.62) |
| 25,000 - 39,999$ | 0.65 (0.30-1.40) | 0.91 (0.32-2.55) | 0.40 (0.13-1.22) | 0.74 (0.26-2.13) | 0.59 (0.20-1.71) |
| 40,000 - 59,999$ | 0.75 (0.36-1.57) | 1.03 (0.31-3.36) | 0.53 (0.21-1.33) | 0.92 (0.35-2.44) | 0.47 (0.14-1.62) |
| ≥ 60,000 $ | 1.59 (0.81-3.14) | 2.17 (0.66-7.14) | 1.19 (0.53-2.67) | 2.47 (0.89-6.88) | 0.94 (0.39-2.29) |
| School attainment (vs high school not completed) | |  |  |  |  |
| Post-secondary | 0.40 (0.16-0.95)* | 0.67 (0.12-3.72) | 0.35 (0.12-0.99)* | 0.40 (0.08-2.06) | 0.43 (0.15-1.26) |
| High school completed | 0.81 (0.50-1.31) | 0.75 (0.40-1.41) | 0.89 (0.42-1.85) | 0.79 (0.40-1.59) | 0.91 (0.50-1.66) |
| Traditional activities: in the last year | 0.78 (0.44-1.36) | 1.41 (0.58-3.44) | 0.53 (0.25-1.11) | 0.84 (0.39-1.81) | 0.74 (0.34-1.59) |
| Going on the land: often | 0.98 (0.65-1.49) | 1.33 (0.77-2.31) | 0.71 (0.36-1.38) | 0.90 (0.48-1.71) | 1.13 (0.69-1.84) |

OR are adjusted for age categories (16-24, 25-34, 35-49, 50-59, ≥ 60 when relevant) and sex.

Other variables not presented because p-value always > 0.2 (with small OR) are electronic cigarette, allergic sensitization to dust mites, and high blood total IgE.

^A^ Airway obstruction was defined as a FEV_1_/FVC ratio below the lower limit of normal (LLN). Sensitivity analysis with the fixed ratio (0.7) is presented in Supplementary Material.

^B^ The reference category is different for young adults because the number of cases among never smokers was too small to yield reliable estimates.

*p<0.05

### **Comparison of Airway Obstruction Defined by LLN Versus Fixed Ratio**

In the previous results, airway obstruction was defined as an FEV_1_/FVC ratio below the lower limit of normal (LLN). C5 compares the prevalence of airway obstruction defined by the fixed ratio versus by the LLN. Using the fixed ratio did not change the prevalence in the total population but changed the prevalence across age groups and sexes, because the fixed ratio does not vary with age or sex. Airway obstruction became less frequent among young adults and more frequent among adults, and the difference between the sexes increased. Tables C6 and C7 respectively present multivariate and age/sex-adjusted models for airway obstruction defined by fixed ratio. There were few significant associations. Airway obstruction tended to be associated with current smoking (≥ 15 pack-years). The main difference was an association with second-hand smoke exposure, especially among adults and men.

Table C5. Prevalence of airway obstruction, defined by lower limit of normal (LLN) and fixed ratio, population of Nunavik aged 16 and over, 2017

|  | Airway obstruction defined by LLN | | Airway obstruction defined by fixed ratio | |
| --- | --- | --- | --- | --- |
|  | N | % (95% CI) | N | % (95% CI) |
| Total population | 176 | 16.8 (14.0-19.6) | 155 | 15.4 (12.6-18.1) |
| Men | 79 | 19.5* (14.8-24.3) | 89 | 21.2* (16.4-26.0) |
| Women | 97 | 14.0* (11.1-16.9) | 66 | 9.3* (6.9-11.7) |
| 16-34 years | 93 | 17.0 (13.2-20.9) | 37 | 8.2* (5.1-11.3) |
| ≥35 years | 83 | 16.6 (12.5-20.7) | 118 | 22.9* (18.4-27.3) |

Numbers (N) of cases are unweighted to represent the sample while proportions (%) are weighted to represent Nunavik’s population.

* p<0.05 for the difference between age groups and the difference between sexes (bilateral Wald chi-square test).

Table C6. Multivariable logistic models for airway obstruction defined by fixed ratio, population of Nunavik aged 16 and over, 2017

|  | Total population  (N = 1,088) | 35 years and older  (N = 553) | Men  (N = 376) | Women  (N = 712) |
| --- | --- | --- | --- | --- |
|  | OR (95% CI) | OR (95% CI) | OR (95% CI) | OR (95% CI) |
| Sex: men versus women | 1.41 (0.57-3.50) | 2.44 (1.37-4.32)* |  |  |
| Tobacco smoking (vs never smoking) |  |  |  |  |
| Former | 0.93 (0.37-2.39) | 0.86 (0.31-2.38) | 1.04 (0.33-3.21) | 0.47 (0.08-2.66) |
| Current, < 15 pack year | 0.72 (0.32-1.60) | 0.77 (0.27-2.20) | 0.69 (0.23-2.11) | 1.06 (0.30-3.69) |
| Current, ≥15 pack year | 2.03 (0.89-4.65) | 2.26 (0.91-5.57) | 2.08 (0.71-6.10) | 2.43 (0.73-8.06) |
| Marijuana use: daily/regular (> once/month) | 0.98 (0.59-1.61) | 0.96 (0.51-1.79) | NS | NS |
| Second-hand smoke exposure: almost daily | 1.74 (1.05-2.89)* | 2.17 (1.18-3.97)* | 2.41 (1.20-4.84)* | 0.89 (0.42-1.85) |
| Body mass index (vs 18.5-24.9) |  |  |  |  |
| < 18,5 (underweight) | 2.17 (0.47-9.95) | 1.01 (0.18-5.61) | 1.78 (0.08-37.83) | 2.28 (0.36-14.63) |
| 25-30 (overweight) | 0.88 (0.48-1.64) | 0.82 (0.39-1.71) | 0.85 (0.35-2.09) | 0.82 (0.37-1.81) |
| ≥ 30 (obesity) | 0.63 (0.31-1.26) | 0.75 (0.34-1.67) | 0.65 (0.25-1.68) | 0.49 (0.20-1.21) |
| Marine mammals & fish (vs < 3 times/week) | |  |  |  |
| 3-7 times/week | NS | NS | 0.81 (0.32-2.10) | 2.87 (1.21-6.77)* |
| ≥7 times/week | NS | NS | 1.05 (0.46-2.40) | 1.29 (0.55-3.02) |
| Fruits/Vegetables: ≥ 5 times/day | NS | NS | 1.37 (0.51-3.71) | 0.39 (0.11-1.30) |
| Vitamin D: sufficient blood level | Ns | 0.64 (0.30-1.37) | NS | NS |
| Respiratory infection during childhood | 1.65 (0.70-3.89) | 1.98 (0.79-5.00) | 1.57 (0.44-5.62) | 1.83 (0.47-7.09) |
| Allergic sensitization to dogs | 2.19 (0.75-6.34) | 3.33 (0.97-11.46) | 1.82 (0.47-7.09) | 3.1 (0.64-14.94) |
| Housing crowding (> 1 person-per-room) | 0.66 (0.37-1.18) | 0.55 (0.26-1.14) | 0.63 (0.29-1.37) | 0.79 (0.34-1.83) |
| Housing in need of major repairs | NS | NS | 0.55 (0.25-1.21) | 1.01 (0.44-2.34) |
| Food security (ref: severely food insecure) | |  |  |  |
| Food secure | NS | 1.4 (0.64-3.04) | 1.48 (0.62-3.56) | 0.82 (0.32-2.09) |
| Moderately food insecure | NS | 0.75 (0.35-1.64) | 0.85 (0.35-2.08) | 1.46 (0.60-3.51) |
| School attainment (ref: high school not completed) | |  |  |  |
| Post-secondary | NS | 0.85 (0.33-2.20) | NS | NS |
| High school completed | NS | 0.8 (0.38-1.70) | NS | NS |
| Traditional activities: in the last year | NS | 1.03 (0.43-2.44) | 1.57 (0.61-4.02) | 0.47 (0.18-1.19) |

Models were not elaborated for young adults (16-34 years old) because there were few cases of obstruction. The fixed ratio underestimates obstruction among younger population and its recommendation as a diagnostic criterion for COPD was not intended to younger populations.

Models include age categories (16-24, 25-34, 35-49, 50-59, ≥ 60) and all variables that are listed in the column, except if indicated as “NS” for “not selected”.

OR are adjusted for age categories (16-24, 25-34, 35-49, 50-59, ≥ 60 when relevant) and all variables in the Table, unless specified as not selected (NS).

* p<0.05

Table C7. Age/sex-adjusted logistic models for airway obstruction, defined by fixed ratio population of Nunavik aged 16 and over, 2017

|  | Total population  (N = 1,088) | 35 years and older  (N = 553) | Men  (N = 376) | Women  (N = 712) |
| --- | --- | --- | --- | --- |
|  | OR (95% CI) | OR (95% CI) | OR (95% CI) | OR (95% CI) |
| Sex: men versus women | 3.02 (1.99-4.59)* | 2.66 (1.61-4.41)* |  |  |
| Tobacco smoking (vs never smoking) |  |  |  |  |
| Former | 0.99 (0.38-2.54) | 0.96 (0.36-2.58) | 1.31 (0.40-4.26) | 0.58 (0.12-2.71) |
| Current, < 15 pack year | 0.94 (0.45-1.95) | 0.95 (0.38-2.38) | 0.82 (0.33-2.02) | 1.42 (0.44-4.58) |
| Current, ≥15 pack year | 2.69 (1.32-5.50)* | 2.71 (1.24-5.92)* | 2.47 (0.99-6.18) | 4.06 (1.39-11.91)* |
| Recreational solvent: ever, at least once | 1.03 (0.62-1.72) | 0.93 (0.52-1.66) | 1.11 (0.58-2.13) | 0.85 (0.42-1.74) |
| Marijuana use: daily/regular (> once/month) | 1.38 (0.87-2.19) | 1.26 (0.72-2.21) | 1.43 (0.77-2.67) | 1.35 (0.73-2.48) |
| Second-hand smoke exposure: almost daily | 2.12 (1.33-3.39)* | 2.35 (1.34-4.10)* | 2.37 (1.26-4.47)* | 1.59 (0.85-2.95) |
| Body mass index (vs 18.5-24.9) |  |  |  |  |
| < 18,5 (underweight) | 2.83 (0.66-12.18) | 1.45 (0.30-7.04) | 1.22 (0.04-40.42) | 3.76 (1.06-13.39)* |
| 25-30 (overweight) | 0.83 (0.47-1.46) | 0.72 (0.39-1.34) | 0.86 (0.41-1.82) | 0.77 (0.37-1.62) |
| ≥ 30 (obesity) | 0.51 (0.27-0.97)* | 0.56 (0.27-1.16) | 0.62 (0.27-1.43) | 0.37 (0.16-0.84)* |
| Marine mammals & fish (vs < 3 times/week) | |  |  |  |
| 3-7 times/week | 1.22 (0.66-2.26) | 1.40 (0.69-2.85) | 0.76 (0.33-1.74) | 2.78 (1.24-6.20) |
| ≥7 times/week | 1.27 (0.71-2.27) | 1.52 (0.79-2.95) | 1.31 (0.62-2.76) | 1.14 (0.51-2.54) |
| Fruits/Vegetables: ≥ 5 times/day | 1.00 (0.47-2.17) | 1.35 (0.55-3.33) | 1.52 (0.59-3.90) | 0.35 (0.10-1.22) |
| Vitamin D: sufficient blood level | 0.83 (0.45-1.52) | 0.59 (0.27-1.27) | 0.84 (0.40-1.77) | 0.83 (0.35-1.96) |
| Respiratory infection during childhood | 1.72 (0.77-3.88) | 1.79 (0.74-4.34) | 1.42 (0.45-4.52) | 2.08 (0.76-5.70) |
| Active TB in the past | 1.07 (0.46-2.51) | 1.11 (0.44-2.77) | 0.70 (0.21-2.33) | 1.75 (0.66-4.65) |
| Allergic sensitization to dogs | 2.04 (0.80-5.19) | 2.48 (0.83-7.40) | 1.60 (0.46-5.48) | 3.03 (0.79-11.55) |
| Housing crowding (> 1 person-per-room) | 0.68 (0.39-1.20) | 0.55 (0.28-1.05) | 0.59 (0.28-1.26) | 0.90 (0.45-1.81) |
| Housing in need of major repairs | 0.69 (0.39-1.23) | 0.82 (0.43-1.60) | 0.56 (0.27-1.18) | 1.06 (0.49-2.28) |
| Food security (vs severely food insecure) | |  |  |  |
| Food secure | 0.93 (0.49-1.73) | 0.86 (0.42-1.77) | 1.16 (0.52-2.58) | 0.60 (0.26-1.41) |
| Moderately food insecure | 1.00 (0.56-1.77) | 0.69 (0.35-1.35) | 0.91 (0.43-1.92) | 1.18 (0.54-2.57) |
| Personal income (vs < 15,000$) |  |  |  |  |
| ≥ 60,000 $ | 1.02 (0.50-2.06) | 0.89 (0.42-1.90) | 1.11 (0.41-3.03) | 0.91 (0.38-2.18) |
| 40,000 - 59,999$ | 0.54 (0.25-1.16) | 0.61 (0.26-1.39) | 0.58 (0.23-1.47) | 0.39 (0.10-1.47) |
| 25,000 - 39,999$ | 0.64 (0.28-1.43) | 0.38 (0.15-0.95)* | 0.69 (0.25-1.88) | 0.60 (0.17-2.10) |
| 15,000 - 24,999$ | 1.10 (0.58-2.11) | 0.60 (0.27-1.31) | 0.95 (0.39-2.32) | 1.50 (0.66-3.41) |
| School attainment (vs high school not completed) | | |  |  |
| Post-secondary | 0.68 (0.32-1.47) | 0.74 (0.34-1.65) | 1.13 (0.36-3.56) | 0.40 (0.13-1.20) |
| High school completed | 0.63 (0.36-1.12) | 0.74 (0.37-1.49) | 0.63 (0.31-1.29) | 0.72 (0.32-1.67) |
| Traditional activities: in the last year | 0.85 (0.46-1.58) | 0.68 (0.32-1.45) | 1.06 (0.50-2.23) | 0.44 (0.19-1.04) |
| Going on the land: often | 1.10 (0.69-1.75) | 0.95 (0.54-1.66) | 1.18 (0.63-2.20) | 0.92 (0.51-1.67) |

Models were not elaborated for young adults (16-34 years old) because there were few cases of obstruction. The fixed ratio underestimates obstruction among younger population and its recommendation as a diagnostic criterion for COPD was not intended to younger populations.

OR are adjusted for age categories (16-24, 25-34, 35-49, 50-59, ≥ 60 when relevant) and sex.

Other variables not presented because p-value always > 0.2 (with small OR) are electronic cigarette, allergic sensitization to dust mites, and high blood total IgE

*p<0.05

### **Post-hoc analyses of marine mammals consumption and fish consumption**

Table C8. Distribution of the new separated variables

|  | Total population | | 16-34 years old | | 35 years and older | | Men | | Women | |
| --- | --- | --- | --- | --- | --- | --- | --- | --- | --- | --- |
|  | N | % | N | % | N | % | N | % | N | % |
| Marine mammals |  |  |  |  |  |  |  |  |  |  |
| ≥7 times/week | 290 | 23.9 | 133 | 22.7 | 157 | 25.2 | 124 | 27.6 | 167 | 20.0 |
| 3-7 times/week | 230 | 17.8 | 117 | 19.8 | 113 | 15.8 | 81 | 18.3 | 149 | 17.3 |
| < 3 times/week | 776 | 58.3 | 379 | 57.5 | 397 | 59.0 | 249 | 54.0 | 527 | 62.7 |
| Fish |  |  |  |  |  |  |  |  |  |  |
| ≥7 times/week | 209 | 17.4 | 96 | 17.0 | 113 | 17.8 | 87 | 19.3 | 122 | 15.4 |
| 3-7 times/week | 204 | 16.4 | 97 | 16.3 | 106 | 16.6 | 77 | 17.4 | 127 | 15.4 |
| < 3 times/week | 883 | 66.2 | 435 | 66.7 | 448 | 65.6 | 289 | 63.3 | 594 | 69.2 |

Data are pooled from multiple imputations. Numbers (N) are rounded and unweighted to represent the sample while proportions (%) are weighted to represent Nunavik’s population. No statistical comparisons were performed between groups.

Table C9. Relation between wheezing and marine mammals or fish consumption, in multivariable logistic models

|  | Total population  (N = 1,258) | 16-34 years old  (N = 608) | 35 years and older  (N = 650) | Men  (N = 439) | Women  (N = 819) |
| --- | --- | --- | --- | --- | --- |
|  | OR (95% CI) | OR (95% CI) | OR (95% CI) | OR (95% CI) | OR (95% CI) |
| Marine mammals (vs < 3 times/week) | | |  |  |  |
| ≥7 times/week |  | 0.73 (0.38-1.42) | 1.66 (0.95-2.90) | 1.21 (0.64-2.89) | 1.41 (0.85-2.34) |
| 3-7 times/week | 1.36 (0.91-2.05) | 1.25 (0.69-2.27) | 1.42 (0.75-2.65) | 1.18 (0.59-2.36) | 1.52 (0.93-2.48) |
| Fish (vs < 3 times/week) |  |  |  |  |  |
| ≥7 times/week | 1.45 (0.95-2.22) | 1.48 (0.71-3.06) | 1.49 (0.82-2.70) | 1.89 (0.97-3.68) | 1.08 (0.62-1.89) |
| 3-7 times/week | 1.35 (0.88-2.06) | 2.50 (1.32-4.72)* | 0.90 (0.47-1.72) | 1.63 (0.83-3.17) | 1.14 (0.67-1.95) |

This table presents the same models as the Table 3, but the variable “Marine mammals and fish” is separated in two variables. The odd ratio of “Marine mammals” and “Fish” are still adjusted for the same covariables than the models in the Table 3, which can vary between groups (total, young adults, adults, men, women).

To avoid redundancy, the odd ratios of other covariables are not presented again. Unless specified as “not selected” in Table 3, the covariables are: age, sex, tobacco smoking, solvent inhalation, marijuana use, second-hand smoke exposure, body mass index, fruits/vegetables, vitamin D, respiratory infection during childhood, allergic sensitization to dog, high blood IgE, housing crowding, housing in need of major repairs, food security, personal income, school attainment, traditional activities, going on the land.

*p<0.05

Table C10. Relation between chronic cough and marine mammals or fish consumption, in multivariable logistic models

|  | Total population  (N= 1,257) | 16-34 years old  (N = 609) | 35 years and older  (N = 648) | Men  (N = 437) | Women  (N = 820) |
| --- | --- | --- | --- | --- | --- |
|  | OR (95% CI) | OR (95% CI) | OR (95% CI) | OR (95% CI) | OR (95% CI) |
| Marine mammals (vs < 3 times/week) | | |  |  |  |
| ≥7 times/week | 1.40 (0.88-2.22) | 2.45 (1.19-5.03)* | 1.08 (0.59-1.97) | 1.11 (0.53-2.32) | 1.76 (1.01-3.07)* |
| 3-7 times/week | 1.18 (0.72-1.93) | 1.57 (0.72-3.43) | 1.09 (0.56-2.14) | 1.07 (0.44-2.61) | 1.21 (0.68-2.16) |
| Fish (vs < 3 times/week) |  |  |  |  |  |
| ≥7 times/week | 1.56 (0.97-2.51) | 1.73 (0.79-3.78) | 1.54 (0.84-2.84) | 3.04 (1.38-6.68)* | 0.95 (0.53-1.72) |
| 3-7 times/week | 1.29 (0.79-2.11) | 1.61 (0.75-3.46) | 1.14 (0.57-2.29) | 1.69 (0.70-4.10) | 1.12 (0.62-2.01) |

This table presents the same models as the Table 4, but the variable “Marine mammals and fish” is separated in two variables. The odd ratio of “Marine mammals” and “Fish” are still adjusted for the same covariables than the models in the Table 4, which can vary between groups (total, young adults, adults, men, women).

To avoid redundancy, the odd ratios of other covariables are not presented again. Unless specified as “not selected” in Table 4, the covariables are: age, sex, tobacco smoking, electronic cigarette, solvent inhalation, marijuana use, second-hand smoke exposure, body mass index, fruits/vegetables, respiratory infection during childhood, active TB in the past, allergic sensitization to dog, housing crowding, housing in need of major repairs, food security, personal income, school attainment, traditional activities, going on the land.

*p<0.05

Table C11. Relation between wheezing and marine mammals or fish consumption, in age/sex-adjusted logistic models

|  | Total population  (N = 1,258) | 16-34 years old  (N = 608) | 35 years and older  (N = 650) | Men  (N = 439) | Women  (N = 819) |
| --- | --- | --- | --- | --- | --- |
|  | OR (95% CI) | OR (95% CI) | OR (95% CI) | OR (95% CI) | OR (95% CI) |
| Marine mammals (vs < 3 times/week) | | |  |  |  |
| ≥7 times/week | 1.50 (1.04-2.15)* | 1.17 (0.67-2.06) | 1.84 (1.14-2.99)* | 1.45 (0.84-2.51) | 1.53 (0.98-2.38) |
| 3-7 times/week | 1.38 (0.93-2.04) | 1.46 (0.84-2.53) | 1.26 (0.73-2.16) | 1.16 (0.62-2.18) | 1.65 (1.04-2.60)* |
| Fish (vs < 3 times/week) |  |  |  |  |  |
| ≥7 times/week | 1.37 (0.92-2.05) | 1.39 (0.76-2.55) | 1.34 (0.79-2.29) | 1.58 (0.86-2.90) | 1.15 (0.71-1.88) |
| 3-7 times/week | 1.42 (0.95-2.12) | 2.12 (1.21-3.73)* | 0.93 (0.53-1.62) | 1.36 (0.73-2.54) | 1.55 (0.96-2.51) |

OR are adjusted for age categories (16-24, 25-34, 35-49, 50-59, ≥ 60 when relevant) and sex.

*p<0.05

Table C12. Relation between chronic cough and marine mammals or fish consumption, in age/sex-adjusted logistic models

|  | Total population  (N= 1,257) | 16-34 years old  (N = 609) | 35 years and older  (N = 648) | Men  (N = 437) | Women  (N = 820) |
| --- | --- | --- | --- | --- | --- |
|  | OR (95% CI) | OR (95% CI) | OR (95% CI) | OR (95% CI) | OR (95% CI) |
| Marine mammals (vs < 3 times/week) | | |  |  |  |
| ≥7 times/week | 1.74 (1.17-2.58)* | 2.63 (1.46-4.75)* | 1.34 (0.80-2.22) | 1.67 (0.88-3.17) | 1.76 (1.11-2.80)* |
| 3-7 times/week | 1.33 (0.85-2.09) | 1.51 (0.75-3.03) | 1.30 (0.72-2.34) | 1.28 (0.58-2.79) | 1.38 (0.83-2.30) |
| Fish (vs < 3 times/week) |  |  |  |  |  |
| ≥7 times/week | 1.43 (0.92-2.23) | 2.05 (1.01-4.13)* | 1.19 (0.68-2.08) | 2.17 (1.07-4.40)* | 1.01 (0.60-1.69) |
| 3-7 times/week | 1.49 (0.95-2.33) | 2.01 (1.06-3.82)* | 1.23 (0.67-2.26) | 1.57 (0.74-3.33) | 1.50 (0.90-2.49)* |

OR are adjusted for age categories (16-24, 25-34, 35-49, 50-59, ≥ 60 when relevant) and sex.

*p<0.05
